# Palladium-catalyzed regio- and stereoselective synthesis of aryl and 3-indolyl-substituted 4-methylene-3,4-dihydroisoquinolin-1(*2H*)-ones

**DOI:** 10.3762/bjoc.16.95

**Published:** 2020-05-20

**Authors:** Valeria Nori, Antonio Arcadi, Armando Carlone, Fabio Marinelli, Marco Chiarini

**Affiliations:** 1 Dipartimento di Scienze Fisiche e Chimiche, Università di L’Aquila, Via Vetoio, 67010 Coppito (AQ), Italy; 2 Facoltà di Bioscienze e Tecnologie Agro-alimentari e Ambientali, Università di Teramo, Via Balzarini 1, 64100 Teramo (Te), Italy

**Keywords:** alkynylanilines, arylboronic acids, indoles, isoquinolinones, palladium

## Abstract

Cascade cyclocarbopalladation of the readily available aryl/alkyl-substituted propargylic amides containing an aryl iodide moiety, followed by Suzuki–Miyaura coupling with arylboronic acids, allowed an efficient regio- and stereoselective synthesis of tetrasubstituted 4-methylene-3,4-dihydroisoquinolin-1(2*H*)-ones. Moreover, cascade cyclocarbopalladation, followed by the reaction with 2-alkynyltrifluoroacetanilides, accomplished a double cyclization to afford challenging 4-methylene-3,4-dihydroisoquinolin-1(2*H*)-ones bearing a 3-indolyl substituent through aminopalladation/reductive elimination.

## Introduction

The isoquinolinone nucleus is a key constituent of many natural products [1–3] and pharmaceuticals [4–6]. Substituted isoquinolinones have been found in biologically active small molecules that exhibit antihypertensive activity [7–8]. Moreover, these heterocycles can be used as 5-HT3 antagonists [9], rho kinase inhibitors [10], thymidylate synthetase inhibitors [11], PARP-1 inhibitors [12], melatonin MT_1_ and MT_2_ receptor agonist [13], and fascin-targeted antimetastatic agents [14]. Fittingly, the development of efficient strategies for their construction and peripheral functionalization represents still an active research area aimed to achieve structural diversity [15–18].

Carbometalations of alkynes constitute a powerful tool for the regio- and stereoselective formation of carbon–carbon bonds [19]. Intramolecular palladium-catalyzed versions are particularly attractive, since they afford polycarbo- and heterocyclic systems via sequential reactions of the vinylpalladium intermediate [20–25]. In this field, a variety of regio- and stereoselective Pd-catalyzed cascade reactions, consisting of the addition of in situ-generated arylpalladium complexes over a proximate carbon–carbon triple bond, followed by cross-coupling reactions, have been reported [26–31].

**Scheme 1:**
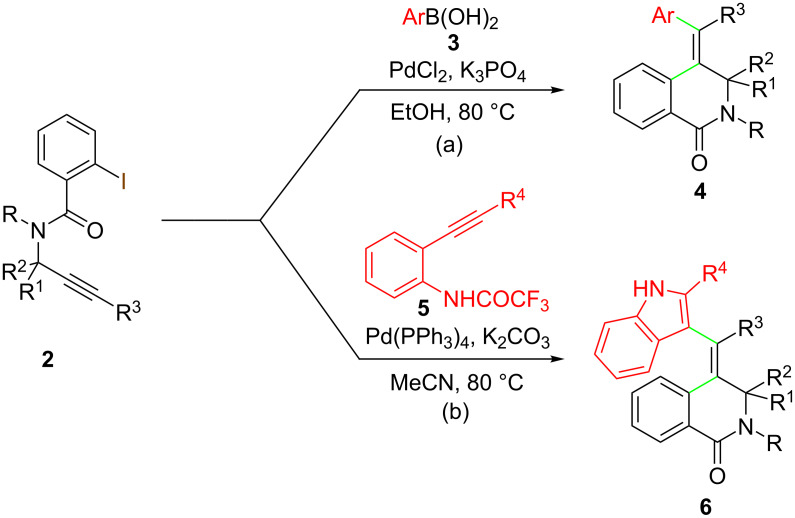
Planned approach to tetrasubstituted-4-methylene-3,4-dihydroisoquinolin-1*(2H*)-ones **4** and **6**.

Our continuing interest in the palladium-catalyzed reactions of functionalized alkynes with boronic acids [32–33] prompted us to explore the palladium-catalyzed reaction of the readily available alkynyliodobenzamides **2** with boronic acids **3** as a viable route to the regio- and stereoselective synthesis of 4-alkylidene-3,4-dihydroisoquinolin-1(2*H*)-ones **3** (Scheme 1a).

We are pleased to report here that this cascade reaction takes place efficiently, resulting in the regio- and stereoselective formation of the poly-substituted isoquinolinones **4** in good to high yield. Applications of this reaction can be relevant for improvements of structure diversity and fine tuning of the chemical and physical properties of the products.

Furthermore, over the years, we have reported a general methodology for the Pd-catalyzed synthesis of 3-substituted indoles, now referred to as the “Cacchi reaction” [34], through an aminopalladation/reductive elimination sequence starting from 2-alkynyltrifluoroacetanilides. In all these procedures, the activation of the triple bond was achieved by means of a σ-organyl palladium complex, in turn generated in situ by oxidative addition of a Pd(0) species to suitable organic electrophiles (aryl and vinyl halides or triflates [35–36], alkyl halides [37], alkynyl halides [38], α-iodoenones [39], or by transmetalation of a Pd(II) species with boronic acids [33]. In this context, we decided to explore the use of substrates **2** in the reaction with 2-alkynyltrifluoroacetanilides **5** through a sequential cyclocarbopalladation/aminopalladation/reductive elimination process, widening in such a way the scope of the methodology and allowing challenging synthesis of indoles **6** bearing a 4-alkylidene-3,4-dihydroisoquinolin-1(2*H*)-one substituent (Scheme 1b). It is worth noting that an aerobic Pd/Cu-catalyzed cyclizative cross-coupling between 2-alkynylanilines and 2-alkynylbenzamides, affording indoles bearing an alkylidene-iminoisobenzofurane moiety, has been reported [40].

## Results and Discussion

**Scheme 2:**
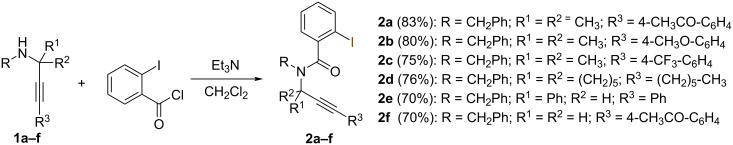
Preparation of the starting *N*-propargyl-2-iodobenzamides **2**.

The starting *N*-propargyl-2-iodobenzamides **2** were easily obtained by the reaction of the readily available [41] propargylamines **1** with 2-iodobenzoyl chloride in CH_2_Cl_2_ at room temperature (Scheme 2).

Initially, we explored the reaction of the *N*-(4-(4-acetylphenyl)-2-methylbut-3-yn-2-yl)-*N*-benzyl-2-iodobenzamide (**2a**) with a variable excess of the phenylboronic acid (**3a**) in the presence of K_3_PO_4_ as the base (K_3_PO_4_: 3 equiv) by using 5 mol % of different palladium catalysts/solvent/temperature combinations. The results are reported in Table 1.

**Table 1:** Optimization of the reaction of propargyl 2-iodobenzamide **2a** with phenylboronic acid (**3a**).^a^

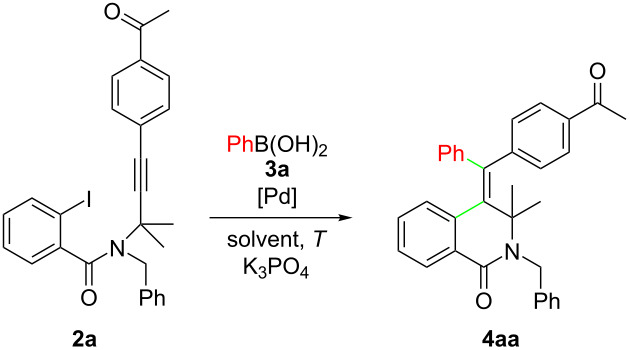					

entry	solvent/temp. (°C)	**3a**:**2a** ratio	catalyst	time (h)	**4aa** yield (%)^b^

1	dioxane/100	1.5	PdCl_2_(PPh_3_)_2_	7	51
2	dioxane/100	2.0	PdCl_2_(PPh_3_)_2_	2	94
3	dioxane/100	3.0	PdCl_2_(PPh_3_)_2_	2	97
4	dioxane/100	1.5	PdCl_2_(PPh_3_)_2_	4	67^c^
5	dioxane/water (9:1)/100	1.5	PdCl_2_(PPh_3_)_2_	2	80^c^
6	MeCN/80	1.5	PdCl_2_(PPh_3_)_2_	7	41^c^
7	THF/60	1.5	PdCl_2_(PPh_3_)_2_	7	27^c^
8	DMF/110	1.5	PdCl_2_(PPh_3_)_2_	2.5	58^c^
9	DMSO/110	1.5	PdCl_2_(PPh_3_)_2_	2.5	41^c^
10	EtOH/80	1.5	PdCl_2_(PPh_3_)_2_	3	85^c^
11	EtOH/80	1.5	Pd(PPh_3_)_4_	3	74^c^
12	EtOH/80	1.5	Pd/C	3	66^c^
13	EtOH/80	1.5	Pd(OAc)_2_	2.5	77^c^
**14**	**EtOH/80**	**1.5**	**PdCl_2_**	**2.5**	**91^c^**

^a^Reactions were carried out on a 0.19 mmol scale, using 3 equiv of base, 0.10 equiv of ligand and 0.05 equiv of the palladium catalyst in 2.0 mL of solvent under nitrogen atmosphere. ^b^Yields are given for isolated products. ^c^1.0 mL of solvent.

When 1,4-dioxane was used as the solvent in the presence of commercially available PdCl_2_(PPh_3_)_2_ as the catalyst at 100 °C, the reaction of **2a** with 1.5 equiv of the phenylboronic acid (**3a**) delivered the target (*Z*)-dihydroisoquinolin-1(*2H*)-one **4aa** in 51% yield. Better yields were observed by increasing the excess of the phenylboronic acid (Table 1, entries 1–3) or by halving the amount of the solvent in the presence 1.5 equiv of **3a** (Table 1, entry 4). Under these latter conditions a beneficial effect was obtained by using a 9:1 mixture of 1,4-dioxane/H_2_O as the reaction medium (Table 1, entry 5). While MeCN, DMF, THF and DMSO as solvents gave worse results (Table 1, entries 6–9), the environmentally friendly EtOH proved to be the most efficient reaction medium (Table 1, entry 10). Further attempts to increase the yield of **4aa** by tuning the catalytic system showed that the ligand-free PdCl_2_ was the most effective catalyst (Table 1, entry 14). Other catalysts such as Pd(PPh_3_)_4_, Pd/C or Pd(OAc)_2_ provided inferior results (Table 1, entries 11–13).

**Scheme 3:**
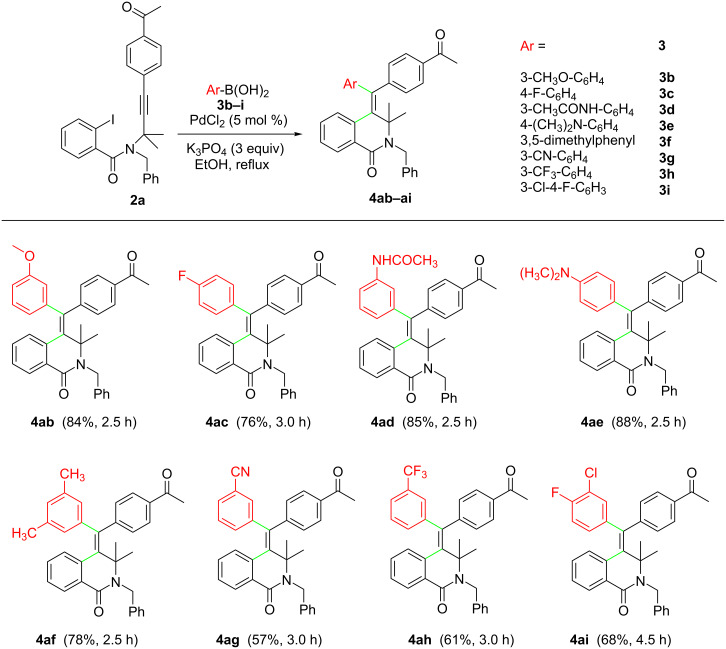
Substrate scope of the reaction of *N*-propargyl-2-iodobenzamide **2a** with arylboronic acids **3b–i**.

We then examined the reaction of **2a** with 1.5 equiv of a variety of arylboronic acids. Using the optimized reaction conditions of Table 1, entry 14, **2a** reacted smoothly with diversely substituted arylboronic acids **3a–i** to regio- and stereoselectively afford the corresponding tetrasubstituted 4-methylene-3,4-dihydroisoquinolin-1(*2H*)-ones **4ab–ai** in moderate to good yields (Scheme 3).

Gratifyingly, various functional groups such as amino, acetamido, F, Cl, OMe, CF_3_, and CN moieties were found to be compatible with these reaction conditions, and only the homocoupling of the arylboronic acids was observed as side reaction to some extent [42]. The best results were obtained with arylboronic acids containing electron-donating substituents; electron-poor arylboronic acids proved to be slightly less effective, probably because of their lower nucleophilicity that could have affected the transmetalation step.

**Scheme 4:**
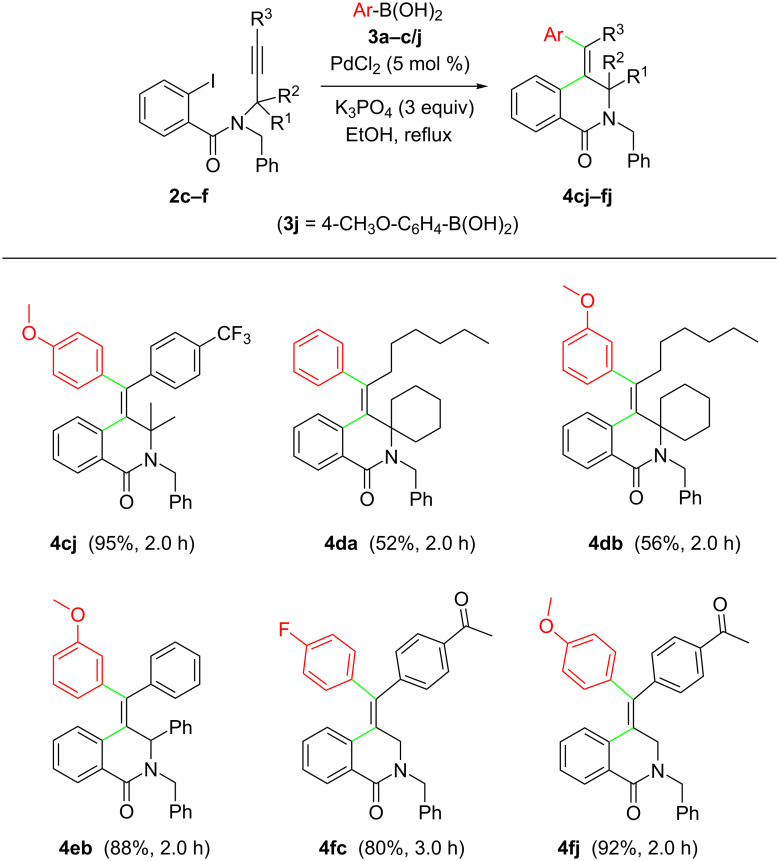
Substrate scope of the reaction of *N*-propargyl-2-iodobenzamides **2c–f** with arylboronic acids **3a–c/j**.

Moreover, we screened the reaction of a number of aromatic boronic acids **3a–j** with a set of *N*-propargyl-2-iodobenzamides **2c–f** (Scheme 4).

All reactions successfully delivered the desired products **4** in moderate to good yields. Remarkably, an alkyl substituent was tolerated at the terminal sp carbon atom of the starting *N*-propargyl-2-iodobenzamides **2**. Furthermore, 2-iodobenzamides **2e** and **2f** (mono-substituted and unsubstituted at the propargylic position) were successfully used as starting materials, affording the corresponding products **4eb**, **4fc** and **4fj** in good yields. According to the literature, the highly stereoselective formation of products **4** resulted from the intramolecular syn-addition of the in situ-generated arylpalladium iodide complex to the triple bond to give an (*E*)-vinylpalladium intermediate, which underwent cross-coupling with an arylboronic acid leading to the final product by reductive elimination, with the regeneration of the Pd(0) catalyst.

**Scheme 5:**
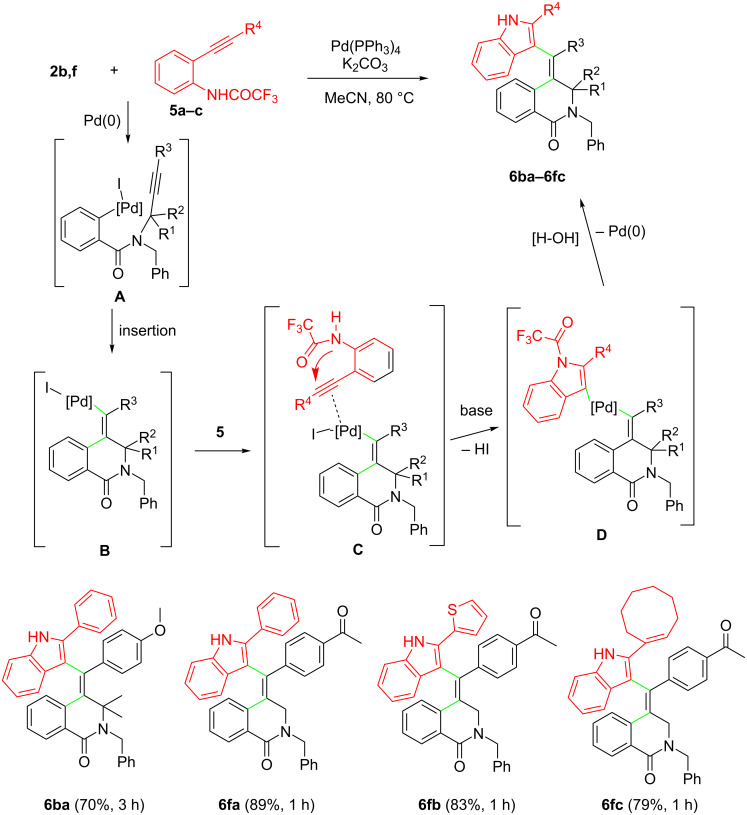
Reaction of *N*-propargyl-2-iodobenzamides **2b,f** with the 2-alkynyltrifluoroacetanilides **5a–c**.

Finally, we envisaged that the above mentioned (*E*)-vinylpalladium intermediate **A** (generated in situ from the insertion of a carbon–carbon triple bond in the initially formed arylpalladium complex) could also be involved in the aminopalladations with the alkynyltrifluoroacetanilides **5** through the formation of the π-complex **B**, followed by base-assisted cyclization and reductive elimination from the resulting σ-indolylpalladium complex **C** (Scheme 5).

The reaction led to the stereoselective formation of indole derivatives **6ba–fc** (aryl, heteroaryl and vinyl groups were allowed in substrates **5**) in good to high yield.

The stereochemistry of compounds **4** and **6** was unambiguously confirmed by NMR spectroscopy [43].

## Conclusion

In conclusion, we have demonstrated that cascade cyclocarbopalladations of the readily available aryl/alkyl-substituted *N*-propargyl-2-iodobenzamides **2** followed by Suzuki–Miyaura coupling reactions with arylboronic acids, in the presence of a catalytic amount of the ligand-free PdCl_2_ in environmentally friendly ethanol, achieve an efficient regio- and stereoselective synthesis of 4-methylene-3,4-dihydroisoquinolin-1(2*H*)-ones **4**. It is worth noting that, during the preparation of this paper, a related article focused on the palladium-catalyzed regioselective cascade cyclization of propargylamides/coupling with ArB(OH)_2_ in dioxane/water, to give trisubstituted arylidene-isoquinolinones [44] was published. However, the Ugi four-component reaction used to construct the starting building blocks was limited to the preparation of propargylic 2-halobenzamides unsubstituted at the propargyl carbon, allowing the synthesis of isoquinolinones without substituents at C-3; but the present methodology overcame these limitations.

Moreover, the previously developed strategy of indole synthesis through an aminopalladation/reductive elimination process has been significantly extended to include σ-vinyl Pd(II) intermediates **B** obtained through oxidative addition/insertion of substrates **2** with Pd(0). This reaction efficiently led to challenging indoloquinolinones **6** through a sequential double cyclization. It is worth noting that, in both cases, the intramolecular alkyne insertion in the initially formed arylpalladium iodide **A** (leading to **B**) occured faster than the direct reaction of **A** with arylboronic acids or with 2-alkynyltrifluoroacetanilides.

## Experimental

### General methods

Melting points are uncorrected. IR spectra were recorded with a Perkin Elmer Spectrum Two FT/IR spectrometer. ^1^H NMR spectra were recorded in CDCl_3_ at 400 MHz on Bruker Avance 400 instrument. Chemical shifts (in ppm) were referenced to tetramethylsilane (δ = 0 ppm) as an internal standard. ^13^C NMR spectra were recorded in CDCl_3_ at 100.6 MHz and were calibrated with CDCl_3_ (δ = 77.00 ppm) or tetramethylsilane (δ = 0 ppm). Mass spectrometry was performed using a MALDI–TOF spectrometer AB SCIEX TOF/TOF 5800 system using 3-hydroxycoumarin or α-cyano-4-hydroxycinnamic acid as a matrix in combination with KI for the ionization. Unless otherwise stated, all starting materials, catalysts, and solvents were commercially available and were used as purchased. The reaction products were purified by flash chromatography on silica gel by elution with *n*-hexane/EtOAc mixtures. Compounds **1a** [44], **1b,c** [41], **1d** [45], **1e** [46] and **5a–c** [47] are known products and were identified by comparison of their physical and spectral data obtained with those reported in the cited references.

### Procedures

**Procedure for the preparation of 1-(4-(3-(benzylamino)prop-1-yn-1-yl)phenyl)ethanone (1f):** To a solution of *N*-benzylprop-2-yn-1-amine (0.25 g, 1,72 mmol) in 3 mL of anhydrous THF were added diisopropylamine (1.2 mL, 8.6 mmol), 4-iodoacetophenone (0.505 g, 2.06 mmol), PdCl_2_(PPh_3_)_2_ (18,1 mg, 0.026 mmol) and CuI (9.8 mg, 0.051 mmol). The mixture was stirred at room temperature under an N_2_ atmosphere for 2 hours. Then the reaction mixture was diluted with ethyl acetate, washed with a solution of NH_4_Cl 0.5 M, dried over Na_2_SO_4_ and concentrated under reduced pressure. The residue was purified by flash chromatography (silica gel, *n*-hexane/EtOAc, 65:35 v/v) to afford 1-(4-(3-(benzylamino)prop-1-yn-1-yl)phenyl)ethanone (**1f**, 385.0 mg, 85%).

**Typical procedure for the preparation of *N*-benzyl-2-iodobenzamides (2a–f):** To a solution (0.25 M) of the propargylamine **1** (1 equiv) [48] in anhydrous dichloromethane (5 mL) were added at room temperature, under N_2_ atmosphere, 2-iodobenzoyl chloride (1.5 equiv) and anhydrous triethylamine (2 equiv). The reaction mixture was stirred under N_2_ until complete consumption of the starting propargylamine (monitored by TLC). Then the reaction mixture was diluted with ethyl acetate, washed with a solution of NH_4_Cl (0.5 M), dried over Na_2_SO_4_ and concentrated under reduced pressure. The residue was purified by flash chromatography (silica gel, *n-*hexane/EtOAc) to afford the *N*-benzyl-2-iodobenzamide **2**.

**Typical procedure for the preparation of-2-benzyl-3,4-dihydroisoquinolin-1(2*H*)-ones (4): preparation of (*Z*)-4-((4-acetylphenyl)(phenyl)methylene)-2-benzyl-3,3-dimethyl-3,4-dihydroisoquinolin-1(2*H*)-one (4aa):** To a stirred solution (0.2 M) of *N*-(4-(4-acetylphenyl)-2-methylbut-3-yn-2-yl)-*N*-benzyl-2-iodobenzamide (**2a**, 100 mg, 0.19 mmol) in EtOH (1 mL), were added phenylboronic acid (**3a**, 34.7 mg, 0.285 mmol) and K_3_PO_4_ (120.9 mg, 0.57 mmol); after 5 min stirring at room temperature, PdCl_2_ (2 mg, 0.0095 mmol) was added. The mixture was stirred at 79 °C under an N_2_ atmosphere and stirring was continued at that temperature until complete consumption of the starting propargylamide **2a** (monitored by TLC). Then the reaction mixture was cooled to room temperature, diluted with ethyl acetate (EtOAc), washed with a solution of NH_4_Cl (0.5 M), dried over Na_2_SO_4_ and concentrated under reduced pressure. The residue was purified by flash chromatography (silica gel, *n*-hexane/EtOAc, 70:30 v/v) to afford the dihydroisoquinolin-1(2*H*)-one **4aa**.

**General procedure for the preparation of indole-substituted dihydroisoquinolin-1(2*H*)-ones 6:** To a stirred solution of propargylamide **2** (0.1 mmol) in MeCN (2 mL) were added 2-alkynyltrifluoroacetylanilide **5** (0.12 mmol) and K_2_CO_3_ (0.3 mmol); after 5 min stirring at room temperature Pd(PPh_3_)_4_ (0.005 mmol) was added. The mixture was stirred at 80 °C under N_2_ atmosphere until complete consumption of the starting propargylamide (monitored by TLC). Then the reaction mixture was cooled to room temperature, diluted with ethyl acetate, washed with a 0.5 M solution of NH_4_Cl, dried over Na_2_SO_4_ and concentrated under reduced pressure. The residue was purified by flash chromatography (silica gel, *n*-hexane/EtOAc, 80:20–50:50 v/v) to afford the desired dihydroisoquinolin-1(2*H*)-one **6**.

## Supporting Information

File 1 Characterization of all new compounds, copies of ^1^H and ^13^C NMR spectra, 2D NOESY experiments.
