# Supplementary material for: Palladium-catalyzed regio- and stereoselective synthesis of aryl and 3-indolyl-substituted 4-methylene-3,4-dihydroisoquinolin-1(2H)-ones
Source: Beilstein J Org Chem. 2020 May 20;16:1084–91. doi: 10.3762/bjoc.16.95 (PMC7277796; doi:10.3762/bjoc.16.95)
Supplement: File 1 — Characterization of all new compounds, copies of 1H and 13C NMR spectra, 2D NOESY experiments. [file Beilstein_J_Org_Chem-16-1084-s001.pdf]

BEILSTEIN JOURNAL OF ORGANIC CHEMISTRY

Supporting Information

for

Palladium-catalyzed regio- and stereoselective synthesis of aryl and 3-indolyl-substituted 4-methylene-3,4-dihydroisoquinolin-1(*2H*)-ones

Valeria Nori, Antonio Arcadi, Armando Carlone, Fabio Marinelli and Marco Chiarini

Beilstein J. Org. Chem. **2020,** *16,* 1084–1091. doi:10.3762/bjoc.16.95

Characterization of all new compounds, copies of ^{1}H and ^{13}C NMR spectra, 2D NOESY experiments

License and Terms: This is a supporting information file under the terms of the Creative Commons Attribution License (http://creativecommons.org/licenses/by/4.0). Please note that the reuse, redistribution and reproduction in particular requires that the authors and source are credited.
The license is subject to the *Beilstein Journal of Organic Chemistry* terms and conditions: (https://www.beilstein-journals.org/bjoc)

Table of Contents

Characterization data of all new compounds…………………………………………………. S2–S7

Copy of ^{1}H NMR and ^{13}C NMR spectra……..…………………………………………………… S8–S34

2D NMR Spectra for the determination of the double bond configuration …………S35–S36

^{1}H NMR and ^{13}C {^{1}H} NMR data

1-(4-(3-(Benzylamino)prop-1-yn-1-yl)phenyl)ethanone (1f): brown oil; AT-IR: 3329, 3028, 2240, 1678, 1601, 1358, 1260, 833, 737, 698 cm^{-1}; ^{1}H NMR (400 MHz, $CDCl_3$) δ = 7.94-7.91 (m, 2H), 7.55-7.52 (m, 2H), 7.41-7.29 (m, 5H), 3.98 (s, 2H), 3.70 (s, 2H), 2.62 (s, 3H) 1.82 (bs, 1H) ppm; ^{13}C NMR (101 MHz, $CDCl_3$) δ = 197.3, 139.3, 136.2, 131.78, 131.77, 131.8, 128.49, 128.48, 128.4, 128.2, 128.1, 127.2, 91.2, 83.1, 52.6, 38.3, 26.6 ppm; HRMS: *m/z* (MALDI-TOF) positive ion, calculated for $C_{18}H_{17}KNO$: $[M+K]^+$ 302.0947, Found: 302.0954.

***N*-(4-(4-Acetylphenyl)-2-methylbut-3-yn-2-yl)-*N*-benzyl-2-iodobenzamide (2a)**: 520.0 mg (83%) as sticky solid; AT-IR: 2233, 1751, 1680, 1641, 1600, 1392, 1357, 1261, 842, 744 cm^{-1}; ^{1}H NMR (400 MHz, $CDCl_3$) δ = 7.84-7.82 (m, 2H), 7.78 (d, *J*=7.9, 1H), 7.34 – 7.27 (m, 2H), 7.26-7.18 (m, 7H), 6.95 (ddd, *J* = 7.9, 6.0, 3.1 Hz, 1H), 4.95 (d, *J* = 17.4 Hz, 1H), 4.53 (d, *J* = 17.4 Hz, 1H), 2.57 (s, 3H), 2.13 (s, 3H), 2.05 (s, 3H) ppm; ^{13}C NMR (101 MHz, $CDCl_3$) δ = 197.3, 171.4, 143.1, 139.0, 138.7, 136.1, 131.6, 130.0, 128.5, 128.2, 128.1, 127.6, 127.05, 127.02, 126.3, 96.1, 92.7, 84.0, 57.1, 52.8, 28.8, 27.6, 26.7 ppm; HRMS: *m/z* (MALDI-TOF) positive ion, calculated for $C_{27}H_{24}IKNO_2$: $[M+K]^+$ 560.0489, Found: 560.0486.

***N*-Benzyl-2-iodo-N-(4-(4-methoxyphenyl)-2-methylbut-3-yn-2-yl)benzamide (2b)**: 489.0 mg (80%) as sticky solid; AT-IR: 2250, 1622, 1605, 1509, 1408, 1154, 972, 834, 764 cm^{-1}; ^{1}H NMR (400 MHz, $CDCl_3$) δ = 7.66 (ddd, *J* = 8.0, 1.0, 0.6 Hz, 1H), 7.20- 7.16 (m, 2H), 7.13-7.08 (m, 3H), 7.06-7.03 (m, 2H), 6.98-6.94 (m, 2H), 6.84 (ddd, *J* = 8.0, 6.6, 2.7 Hz, 1H), 6.67-6.63 (m, 2H), 4.91 (d, *J* = 17.3 Hz, 1H), 4.38 (d, *J* = 17.3 Hz, 1H), 3.66 (s, 3H), 2.00 (s, 3H), 1.95 (s, 3H) ppm; ^{13}C NMR (101 MHz, $CDCl_3$) δ = 171.3, 159.6, 143.6, 139.1, 133.0, 129.8, 128.3, 128.1, 127.1, 126.9, 126.6, 114.8, 113.9, 92.8, 91.4, 85.0, 57.6, 55.3, 53.0, 29.1, 27.7 ppm; HRMS: *m/z* (MALDI-TOF) positive ion, calculated for $C_{26}H_{24}IKNO_2$: $[M+K]^+$ 548.0489, Found: 548.0489.

***N*-Benzyl-2-iodo-N-(2-methyl-4-(4-(trifluoromethyl)phenyl)but-3-yn-2-yl)benzamide (2c)**: 492.0 mg (75%) as sticky solid; AT-IR: 2103, 1652, 1602, 1395, 1319, 1165, 1123, 841, 750, 727 cm^{-1}; ^{1}H NMR (400 MHz, $CDCl_3$) δ = 7.71-7.69 (m, 1H), 7.41-7.38 (m, 2H), 7.24-7.08 (m, 9H), 6.88 (ddd, *J* = 8.0, 6.2, 2.9 Hz, 1H), 4.84 (d, *J* = 17.3 Hz, 1H), 4.43 (d, *J* = 17.3 Hz, 1H), 2.02 (s, 3H) 1.94 (s, 3H) ppm; ^{13}C NMR (101 MHz, $CDCl_3$) δ = 171.3, 143.2, 139.0, 138.8, 131.6, 129.80, 129.75 (q, $^2J_{CF}$ =32.6 Hz), 128.3, 128.0, 126.94, 126.90, 126.5 (q, $^5J_{CF}$ =1.4 Hz), 126.2, 124.9 (q, $^3J_{CF}$ =3.8Hz), 123.8 (q, $^1J_{CF}$ =272.1 Hz), 95.2, 92.5, 83.3, 57.0, 52.7, 28.7, 27.4 ppm; HRMS: *m/z* (MALDI-TOF) positive ion, calculated for $C_{26}H_{21}F_3INNaO_2$: $[M+Na]^+$ 586.0467, Found: 586.0459.

***N*-Benzyl-2-iodo-*N*-(1-(oct-1-yn-1-yl)cyclohexyl)benzamide (2d)**: 520.0 mg (76%) as pale yellow oil; AT-IR: 2927, 2860, 1647, 1392, 1247, 1616, 724, 697 cm^{-1}; ^{1}H NMR (400 MHz, $CDCl_3$) δ = 7.67 (d, *J* = 7.9 Hz, 1H), 7.18-7.14 (m, 2H), 7.11-7.01 (m, 4H), 6.96 (m, 1H), 6.83 (td, *J* = 7.7, 1.6 Hz, 1H), 4.94 (d, *J* = 17.3 Hz, 1H), 4.31 (d, *J* = 17.3 Hz, 1H), 2.95 – 2.70 (m, 2H), 2.01-1.66 (m, 4H), 1.63 – 1.51 (m, 5H), 1.26-1.11 (m, 9H), 0.79 (t, *J* = 7.0 Hz, 3H) ppm; ^{13}C NMR (101 MHz, $CDCl_3$) δ = 171.4, 144.0, 139.6, 138.9, 129.4, 128.0, 127.9, 126.9, 126.6, 126.4, 92.5, 89.4, 80.6, 63.3, 52.7, 34.7, 33.7, 31.3, 28.5, 28.4, 24.9, 24.1, 23.9, 22.5, 18.8, 14.0 ppm; HRMS: *m/z* (MALDI-TOF) positive ion, calculated for $C_{28}H_{34}IKNO$: $[M+K]^+$ 566.1322, Found: 566.1320.

***N*-Benzyl-*N*-(1,3-diphenylprop-2-yn-1-yl)-2-iodobenzamide (2e)**: complex mixture of four slowly interconverting amide geometric 1S,1E/1S,1Z and 1R,1E/1R,1Z isomers; 442.0 mg (70%) as viscous oil; AT-

IR:1639, 1600, 1401, 750, 686 cm^{-1}; ^{1}H NMR (400 MHz, $CDCl_3$) δ = 7.94, 7.92, 7.81, 7.79, 7.64, 7.60, 7.58, 7.54, 7.53, 7.48, 7.47, 7.31, 7.30, 7.25, 7.18, 7.06, 7.04, 6.97, 6.95, 6.91, 6.87, 6.85, 6.80, 6.78, 6.76, 5.79, 5.72, 5.11, 4.91, 4.87, 4.79, 4.76, 4.45, 4.41, 4.35, 4.31, 4.26 ppm; ^{13}C NMR (101 MHz, $CDCl_3$) δ = 171.1, 170.6, 141.7, 141.4, 140.2, 139.92, 139.88, 138.9, 138.0, 137.7, 137.2, 136.3, 135.1, 133.0, 131.7, 131.6, 131.0, 130.6, 130.3, 129.9, 129.8, 129.1, 128.9, 128.7, 128.4, 128.3, 128.2, 127.8, 127.7, 127.54, 127.48, 126.8, 126.7, 122.5, 93.1, 92.3, 88.1, 87.4, 85.3, 56.2, 55.3, 50.2, 49.8, 47.1, 46.4 ppm; HRMS: *m/z* (MALDI-TOF) positive ion, calculated for $C_{29}H_{22}IKNO$: $[M+K]^+$ 566.0383, Found: 566.0385.

***N*-(3-(4-Acetylphenyl)prop-2-yn-1-yl)-*N*-benzyl-2-iodobenzamide (2f)**: mixture of two slowly interconverting geometric *E*/*Z* isomers; 414.0 mg (70%) as viscous oil; AT-IR 3064, 3029, 1681, 1643, 1600, 1414, 1359, 1260, 747, 697 cm^{-1}; ^{1}H NMR (400 MHz, $CDCl_3$) δ = 7.83-7.82 (m, 2H + 2H), 7.77 (ddd, *J*=8.0, 2.4, 0.8 Hz, 1H+1H), 7.46-7.40 (m, 2H + 2H), 7.38-7.21 (m, 6H + 6H), 7.16-7.13 (m, 1H + 1H), 7.04-6.97 (m, 1H + 1H), 5.19 (d, *J*=14.4 Hz, 1H), 4.86 (d, *J*=16.5 Hz, 1H), 4.59 (d, *J*=14.4 Hz, 1H), 4.45 (d, *J*=17.9 Hz, 2H), 4.09 (d, *J*=16.5 Hz, 1H), 3.91 (s, 2H), 2.51 (s, 3H), 2.50 (s, 3H) ppm; ^{13}C NMR (101 MHz, $CDCl_3$) δ = 197.2, 197.1, 170.6, 170.4, 141.6, 141.3, 139.45, 139.36, 136.6, 136.4, 136.0, 135.4, 131.9, 131.8, 130.6, 130.5, 129.0, 128.9, 128.7, 128.44, 128.38, 128.25, 128.23, 128.1, 127.9, 127.7, 127.6, 127.5, 127.3, 127.0, 92.8, 92.5, 87.0, 86.6, 84.3, 83.5, 51.7, 47.5, 38.5, 34.0, 26.6 ppm; HRMS: *m/z* (MALDI-TOF) positive ion, calculated for $C_{25}H_{20}IKNO_2$: $[M+K]^+$ 532.0176, Found: 532.0175.

(*Z*)-4-((4-Acetylphenyl)(phenyl)methylene)-2-benzyl-3,3-dimethyl-3,4-dihydroisoquinolin-1(2*H*)-one (4aa): 82.0 mg (91%) as colourless powder; mp 147-149 °C; AT-IR: 1690, 1640,1594, 1356, 700 cm^{-1}; ^{1}H NMR (400 MHz, $CDCl_3$) δ = 8.00 (dd, *J* = 7.7, 0.9 Hz, 1H), 7.77-7.75 (m, 2H), 7.22-7.11 (m, 8H), 7.01-6.95 (m, 4H), 6.85-6.83 (m, 2H), 6.71 (dd, *J* = 7.8, 0.9 Hz, 1H), 4.88 (bs, 2H), 2.50 (s, 3H), 1.10 (s, 6H) ppm; ^{13}C NMR (101 MHz, CDCl3) δ = 197.5, 165.8, 148.9, 143.7, 139.8, 139.7, 138.4, 137.8, 135.7, 131.1, 131.0, 130.6, 129.5, 128.5, 128.2, 128.1, 127.6, 127.4, 127.1, 126.85, 126.76, 62.9, 45.1, 29.0, 26.6 ppm; HRMS: *m/z* (MALDI-TOF) positive ion, calculated for $C_{33}H_{29}KNO_2$: $[M+K]^+$ 510.1835, Found: 510.1835.

(*E*)-4-((4-Acetylphenyl)(3-methoxyphenyl)methylene)-2-benzyl-3,3-dimethyl-3,4-dihydroisoquinolin-1(2H)-one (4ab): 79.0 mg (84%) as colourless powder; mp 151-153 °C; AT-IR:1685, 1639, 1594, 1271, 710 cm^{-1};^{1}H NMR (400 MHz, $CDCl_3$) δ = 8.00 (dd, *J* = 7.8, 1.4 Hz, 1H), 7.76-7.74 (m, 2H), 7.22-7.19 (m, 8H), 7.00 (td, *J* = 7.6, 1.3 Hz, 1H), 6.91 (t, *J* = 7.9 Hz, 1H), 6.78 (ddd, *J* = 7.8, 1.1, 0.5 Hz, 1H), 6.56 (ddd, *J* = 8.3, 2.6, 0.9 Hz, 1H), 6.44 (ddd, *J* = 7.7, 1.6, 1.0 Hz, 1H), 6.37 (dd, *J* = 2.6, 1.6 Hz, 1H), 4.88 (bs, 2H), 3.50 (s, 3H), 2.50 (s, 3H), 1.09 (s, 6H) ppm; ^{13}C NMR (101 MHz, $CDCl_3$) δ = 197.5, 165.9, 159.2, 148.8, 144.9, 139.7, 139.6, 138.5, 137.8, 135.8, 130.9, 130.7, 129.5, 129.1, 128.5, 128.2, 127.7, 127.4, 126.9, 126.8, 123.6, 116.7, 112.6, 62.9, 55.2, 45.1, 29.0, 26.6 ppm; HRMS: *m/z* (MALDI-TOF) positive ion, calculated for $C_{34}H_{31}NNaO_3$: $[M+Na]^+$ 524.2202, Found: 524.2210.

(*E*)-4-((4-Acetylphenyl)(4-fluorophenyl)methylene)-2-benzyl-3,3-dimethyl-3,4-dihydroisoquinolin-1(2*H*)-one (4ac): 71.0 mg (76%) as colourless powder; mp 145-147 °C; AT-IR: 1685, 1640, 1599, 1503, 1397, 1362, 1261, 837 cm^{-1};^{1}H NMR (400 MHz, $CDCl_3$) δ = 8.00 (ddd, *J* = 7.8, 1.4, 0.5 Hz, 1H), 7.78-7.76 (m, 2H), 7.23-7.19 (m, 5H), 7.16-7.09 (m, 3H), 7.02 (td, *J* = 7.6, 1.4 Hz, 1H), 6.80 (dd, *J* = 9.0, 5.4 Hz, 2H), 6.72-6.67 (m, 3H), 4.87 (bs, 2H), 2.50 (s, 3H), 1.09 (s, 6H) ppm; ^{13}C NMR (101 MHz, $CDCl_3$) δ = 197.4, 165.7, 161.7 (d, $^{1}J_{CF}$ =248.3 Hz), 148.6, 139.7, 139.6, 138.6, 138.23, 138.15 (d, $^{4}J_{CF}$ =0.7 Hz), 135.9, 132.6 (d, $^{3}J_{CF}$ =8.1 Hz), 131.0, 130.7, 130.1,

129.5, 128.4, 128.2, 127.7, 127.5, 126.82, 126.75, 115.2 (d, $^2J_{CF}$ =21.5 Hz), 62.9, 45.1, 28.9, 26.6 ppm; HRMS: *m/z* (MALDI-TOF) positive ion, calculated for $C_{33}H_{28}FKNO_2$: $[M+K]^+$ 528.1741, Found: 528.1738.

(*E*)-*N*-(3-((4-Acetylphenyl)(2-benzyl-3,3-dimethyl-1-oxo-2,3-dihydroisoquinolin-4(1*H*)ylidene)methyl)phenyl)acetamide (4ad): 85.0 mg (85%) as pale yellow powder; mp 163-165 °C; AT-IR: 3266, 1693, 1676, 1623, 1598, 1266, 705 cm^{-1};^{1}H NMR (400 MHz, $CDCl_3$) δ = 7.93 (dd, *J* = 7.8, 1.1 Hz, 1H), 7.73-7.71 (m, 2H), 7.56 (s, 1H), 7.41 (d, *J* = 7.7 Hz, 1H), 7.20-7.08 (m, 7H), 6.99 (td, *J* = 7.6 1.4 Hz, 1H), 6.93 (t, *J* = 7.9 Hz, 1H), 6.88 (s, 1H), 6.78 (d, *J* = 7.7 Hz, 1H), 6.57 (d, *J* = 7.8 Hz, 1H), 4.86 (bs, 2H), 2.47 (s, 3H), 1.96 (s, 3H), 1.08 (s, 6H) ppm;^{13}C NMR (101 MHz, $CDCl_3$) δ = 197.6, 168.5, 166.0, 148.5, 144.3, 139.5, 139.4, 138.3, 138.2, 137.9, 135.8, 131.01, 130.9, 129.8, 129.5, 128.9, 128.5, 128.2, 127.8, 127.3, 126.8, 126.6, 121.8, 118.7, 63.0, 45.1, 28.9, 26.6, 24.5 ppm; HRMS: *m/z* (MALDI-TOF) positive ion, calculated for $C_{35}H_{32}KN_2O_3$: $[M+K]^+$ 567.2050, Found: 567.2052.

(*E*)-4-((4-Acetylphenyl)(4-(dimethylamino)phenyl)methylene)-2-benzyl-3,3-dimethyl-3,4-dihydroisoquinolin-1(2*H*)-one (4ae): 86.0 mg (88%) as yellow powder; mp 179-181; AT-IR: 1739, 1636, 1603, 1519, 1360, 816, 713 cm^{-1}; ^{1}H NMR (400 MHz, $CDCl_3$) δ = 7.98 (ddd, *J* = 7.7, 1.5, 0.5 Hz, 1H), 7.76-7-74 (m, 2H), 7.20-7.09 (m, 8H), 7.03 (td, *J* = 7.6, 1.5 Hz, 1H), 6.82 (ddd, *J* = 7.8, 1.2, 0.6 Hz, 1H), 6.64-6.62 (m, 2H), 6.32-6.29 (m, 2H), 4.88 (bs, 2H), 2.79 (s, 6H), 2.50 (s, 3H), 1.08 (s, 6H) ppm; ^{13}C NMR (101 MHz, $CDCl_3$) δ = 197.6, 166.1, 149.9, 149.3, 140.1, 139.8, 139.5, 135.6, 135.3, 132.4, 131.3, 130.7, 129.89, 129.86, 129.3, 128.4, 128.0, 127.2, 127.0, 126.8, 126.6, 111.5, 63.1, 45.1, 40.2, 28.9, 26.6 ppm; HRMS: *m/z* (MALDI-TOF) positive ion, calculated for $C_{35}H_{34}KN_2O_2$: $[M+K]^+$ 553.2257, Found: 553.2254.

(*E*)-4-((4-Acetylphenyl)(3,5-dimethylphenyl)methylene)-2-benzyl-3,3-dimethyl-3,4-dihydroisoquinolin-1(2*H*)-one (4af): 74.0 mg (78%) as colourless powder; mp 146-149 °C; AT-IR: 1681, 1640, 1599, 1261, 799 cm^{-1}; ^{1}H NMR (400 MHz, $CDCl_3$) δ = 7.99 (ddd, *J* = 7.8, 1.4, 0.5 Hz, 1H), 7.76-7.74 (m, 2H), 7.22-7.11 (m, 8H), 6.98 (td, *J* = 7.6, 1.4 Hz, 1H), 6.74 (ddd, *J* = 7.8, 1.2, 0.6 Hz, 1H), 6.64 (bs, 1H), 6.43 (bs, 2H), 4.87 (bs, 2H), 2.50 (s, 3H), 2.00 (s, 6H), 1.08 (s, 6H) ppm;^{13}C NMR (101 MHz, $CDCl_3$) δ = 197.6, 166.0, 149.1, 143.4, 140.0, 139.7, 138.6, 137.5, 137.2, 135.7, 131.0, 130.5, 129.8, 129.4, 128.9, 128.6, 128.4, 128.1, 127.4, 127.3, 126.9, 126.7, 62.9, 45.2, 29.0, 26.6, 21.2 ppm; HRMS: *m/z* (MALDI-TOF) positive ion, calculated for $C_{35}H_{33}KNO_2$: $[M+K]^+$ 538.2148, Found: 538.2140.

(*E*)-3-((4-Acetylphenyl)(2-benzyl-3,3-dimethyl-1-oxo-2,3-dihydroisoquinolin-4(1*H*)-ylidene)methyl)benzonitrile (4ag): 54.0 mg (57%) as colourless powder; mp 159-161°C; AT-IR: 2228, 1685, 1643, 1597, 1385, 1267, 776, 695 cm^{-1};^{1}H NMR (400 MHz, $CDCl_3$) δ = 8.04 (ddd, *J* = 7.8, 1.4, 0.4 Hz, 1H), 7.80-7.78 (m, 2H), 7.31 (ddd, 6.6, 2.2, 1.6 Hz, 1H), 7.27 – 7.08 (m, 11H), 7.02 (td, *J* = 7.6, 1.4 Hz, 1H), 6.62 (ddd, *J* = 7.8, 1.1, 0.6 Hz, 1H), 4.87 (bs, 2H), 2.52 (s, 3H), 1.10 (s, 6H) ppm; ^{13}C NMR (101 MHz, $CDCl_3$) δ = 197.3, 165.5, 147.5, 145.0, 140.0, 139.5, 137.33, 137.28, 136.3, 135.3, 134.2, 131.05, 130.96, 130.7,130.2, 129.6, 129.0, 128.54, 128.51, 128.4, 127.9, 126.9, 118.3, 112.5, 63.0, 45.2, 29.7, 28.9, 26.6 ppm; HRMS: *m/z* (MALDI-TOF) positive ion, calculated for $C_{34}H_{28}KN_2O_2$: $[M+K]^+$ 535.1788, Found: 535.1787.

(*E*)-4-((4-Acetylphenyl)(3-(trifluoromethyl)phenyl)methylene)-2-benzyl-3,3-dimethyl-3,4-dihydroisoquinolin-1(2*H*)-one (4ah): 62.0 mg (61%) as colourless powder; mp 161-163 °C; AT-IR 1683, 1630, 1597, 1257, 779, 672 cm^{-1};^{1}H NMR (400 MHz, $CDCl_3$) δ = 8.02 (ddd, *J* = 7.8, 1.4, 0.5 Hz, 1H), 7.79-7.77 (m, 2H),

7.28-7.01 (m, 12H), 6.98 (td, *J* = 7.6, 1.4 Hz, 1H), 6.61 (ddd, *J* = 7.8, 1.2, 0.5 Hz, 1H), 4.88 (bs, 2H), 2.51 (s, 3H), 1.10 (s, 6H) ppm; ^{13}C NMR (101 MHz, $CDCl_3$) δ = 196.4, 164.6, 146.8, 143.4, 138.5, 138.4, 137.0, 136.6, 135.1, 133.2 (q, $^4J_{CF}$ =1.2 Hz), 129.9, 129.8, 129.6 (q, $^2J_{CF}$ =32.4 Hz), 129.0, 128.6, 127.6, 127.5, 127.3, 127.1, 126.7, 126.4 (q, $^3J_{CF}$ =3.8 Hz), 125.9, 125.8, 122.8 (q, $^3J_{CF}$ =3.7 Hz), 122.6 (q, $^1J_{CF}$ =272.5 Hz), 61.9, 44.2, 27.9, 25.6 ppm; HRMS: *m/z* (MALDI-TOF) positive ion, calculated for $C_{34}H_{28}F_3KNO_2$: $[M+K]^+$ 578.1709, Found: 578.1715.

(*E*)-4-((4-Acetylphenyl)(3-chloro-4-fluorophenyl)methylene)-2-benzyl-3,3-dimethyl-3,4-dihydroisoquinolin-1(2*H*)-one (4ai): 68.0 mg (68%) as colourless powder; mp 148-150 °C; AT-IR: 1685, 1644, 1599, 1260, 801 cm^{-1}; ^{1}H NMR (400 MHz, $CDCl_3$) δ = 8.03 (dd, *J* = 7.7, 1.1 Hz, 1H), 7.79-7.77 (m, 2H), 7.25 (td, *J* = 7.6, 1.0 Hz, 1H), 7.24 – 7.09 (m, 7H), 7.06 (td, *J* = 7.6, 1.3 Hz, 1H), 6.84 (dd, *J* = 7.0, 2.2 Hz, 1H), 6.76 (t, *J* = 8.5 Hz, 1H), 6.71- 6.67 (m, 2H), 4.87 (bs, 2H), 2.51 (s, 3H), 1.08 (s, 6H) ppm;^{13}C NMR (101 MHz, $CDCl_3$) δ = 196.3, 164.6, 156.0 (d, $^1J_{CF}$ =251.0 Hz), 146.9, 139.7 (d, $^4J_{CF}$ =4.2 Hz), 138.5, 138.1, 136.7, 136.3, 135.1, 131.7, 130.0, 129.9, 129.8 (d, $^3J_{CF}$ =7.1 Hz), 129.0, 128.5, 127.5, 127.4, 127.1, 126.7, 125.9, 125.8, 119.9 (d, $^2J_{CF}$ =18.0 Hz), 115.3 (d, $^2J_{CF}$ =21.2 Hz), 61.9, 44.2, 27.9, 25.6 ppm; HRMS: m/z (MALDI-TOF) positive ion, calculated for $C_{33}H_{27}ClFKNO_2$: $[M+K]^+$ 562.1351, Found: 562.1354.

(*E*)-2-Benzyl-4-((4-methoxyphenyl)(4-(trifluoromethyl)phenyl)methylene)-3,3-dimethyl-3,4-dihydroisoquinolin-1(2*H*)-one (4cj): 85.0 mg (95%) as colourless powder; mp 165-167 °C; AT-IR: 1637, 1594, 1320, 1126, 773, 713, 696 cm^{-1};^{1}H NMR (400 MHz, $CDCl_3$) δ = 8.00 (ddd, *J* = 7.8, 1.5, 0.6 Hz, 1H), 7.42-7.40 (m, 2H), 7.22-7.12 (m, 8H), 7.00 (td, *J* = 7.6, 1.3 Hz, 1H), 6.91 (t, *J* = 8.0 Hz, 1H), 6.78 (ddd, *J* = 7.8, 1.2, 0.6 Hz, 1H), 6.56 (ddd, *J* = 8.3, 2.6, 0.8 Hz, 1H), 6.43 (ddd, *J* = 7.6, 1.6, 0.9 Hz, 1H), 6.36 (dd, *J* = 2.4, 1.7 Hz, 1H), 4.88 (bs, 2H), 3.50 (s, 3H), 1.08 (s, 6H) ppm; ^{13}C NMR (101 MHz, $CDCl_3$) δ = 164.7, 158.2, 146.3 (q, $^5J_{CF}$ = 1.4 Hz), 143.9, 138.7, 138.1, 137.4, 137.1, 129.8, 129.7, 128.8, 128.5, 128.3 (q, $^2J_{CF}$ = 32.4 Hz), 128.1, 127.4, 126.7, 126.4, 125.9, 125.8, 124.0 (q, $^3J_{CF}$ = 3.5 Hz), 123.0 (q, $^1J_{CF}$ = 272.3 Hz), 122.5, 115.7, 111.5, 61.9, 54.1, 44.1, 28.0 ppm; HRMS: *m/z* (MALDI-TOF) positive ion, calculated for $C_{33}H_{28}F_3KNO_2$: $[M+K]^+$ 566.1709, Found: 566.1711.

(*E*)-2'-Benzyl-4'-(1-phenylheptylidene)-2',4'-dihydro-1'*H*-spiro[cyclohexane-1,3'-isoquinolin]-1' (4'*H*)-one (4da): 47.0 mg (52%) as yellow oil; AT-IR 3062, 3031, 1638, 1597, 1454, 1389, 1258, 768, 702 cm^{-1}; ^{1}H NMR (400 MHz, $CDCl_3$) δ = 7.85 (dd, *J* = 7.7, 1.0 Hz, 1H), 7.32-7.30 (m, 3H), 7.25-7.18 (m, 4H), 7.16-7.09 (m, 1H), 7.03 (td, *J* = 7.6, 1.1 Hz, 1H), 6.98 (t, *J* = 8.7 Hz, 1H), 6.81 (td, *J* = 7.6, 1.3 Hz, 1H), 6.31-6.29 (m, 2H), 4.99 (d, *J* = 15.0 Hz, 1H, CH_2 AB system), 4.94 (d, *J* = 15.0 Hz, 1H, CH_2 AB system), 2.81-2.54 (m, 2H), 2.20-1.59 (m, 7H), 1.27-1.17 (m, 4H), 1.11-0.83 (m, 9H), 0.74 (t, *J* = 7.2 Hz, 3H) ppm; ^{13}C NMR (101 MHz, $CDCl_3$) δ = 166.5, 144.9, 142.8, 140.9, 140.1, 131.9, 131.2, 131.1, 130.7, 129.9, 128.9, 128.5, 128.4, 127.5, 126.8, 126.7, 126.0, 125.8, 65.8, 44.7, 39.4, 35.7, 33.0, 31.5, 29.5, 28.1, 25.8, 24.9, 23.8, 22.6, 14.0 ppm; HRMS: *m/z* (MALDI-TOF) positive ion, calculated for $C_{34}H_{39}KNO$: $[M+K]^+$ 516.2669, Found: 516.2667.

(*E*)-2'-Benzyl-4'-(1-(3-methoxyphenyl)heptylidene)-2',4'-dihydro-1'*H*-spiro[cyclohexane-1,3'-isoquinolin]-1'(*4'H*)-one (4db): 54.0 mg (56%) as yellow oil; AT-IR 3067, 3036, 1641, 1599, 1455, 1392, 1283, 1261, 769, 725, 696 cm^{-1}; ^{1}H NMR (400 MHz, $CDCl_3$) δ = 7.86 (bs, 1H), 7.32 – 7.30 (m, 2H), 7.23 (t, *J* = 7.5 Hz, 2H), 7.19 (s, 1H), 7.15 (t, *J* = 7.2 Hz, 1H), 7.05 (t, *J* = 8.2 Hz, 2H), 6.85 (td, *J* = 7.5, 1.0 Hz, 1H), 6.55 (dd, *J* = 8.1, 2.0 Hz, 1H), 6.43-6.40 (m, 1H), 6.37-6.35 (m, 1H), 4.96 (bs, 2H), 3.70 (s, 3H), 2.79-2.55 (m, 2H), 2.18-2.16 (m, 1H), 1.95-1.44 (m, 7H), 1.26-1.00 (m, 10H), 0.75 (t, *J* = 7.2 Hz, 3H) ppm; ^{13}C NMR (101 MHz, $CDCl_3$) δ = 166.5, 161.00, 156.87, 146.26, 140.95, 140.04, 130.56, 130.10, 128.63, 128.47, 127.48, 126.83, 126.72, 126.04, 107.75, 106.37, 101.50, 65.8, 55.2, 44.7, 39.5, 35.7, 33.0, 31.5, 29.5, 28.2, 25.8, 24.9, 23.8, 22.6, 14.0 ppm; HRMS: *m/z* (MALDI-TOF) positive ion, calculated for $C_{35}H_{41}KNO_2$: $[M+K]^+$ 546.2774, Found: 546.2771.

(*Z*)-2-Benzyl-4-((3-methoxyphenyl)(phenyl)methylene)-3-phenyl-3,4-dihydroisoquinolin-1(2*H*)-one (4eb): 84.0 mg (88%) as colourless solid; mp 151-153; AT-IR: 3069, 3034, 1649, 1599, 1262, 762, 696 cm^{-1}; ^{1}H NMR (400 MHz, $CDCl_3$) δ = 8.07 (d, *J* = 7.8 Hz, 1H), 7.40 -7.06 (m, 11H), 7.00-6.96 (m, 2H), 6.91-6.88 (m, 2H), 6.80-6.70 (m, 3H), 6.64-6.47 (m, 4H), 5.65 (d, *J* = 14.8 Hz, 1H), 5.02 (s, 1H), 3.51 (s, 3H), 3.48 (d, *J* = 14.8 Hz, 1H) ppm; ^{13}C NMR (101 MHz, $CDCl_3$) δ = 164.0, 159.4, 142.5, 142.2, 140.7, 139.4, 137.4, 135.8, 130.8, 130.5, 129.7, 129.5, 129.3, 128.8, 128.7, 128.63, 128.60, 128.5, 128.30, 128.27, 128.2, 128.2, 127.9, 127.8, 127.38, 127.36, 127.1, 126.8, 122.7, 115.7, 113.0, 62.0, 55.1, 48.5 ppm; HRMS: *m/z* (MALDI-TOF) positive ion, calculated for $C_{36}H_{29}KNO_2$: $[M+K]^+$ 546.1835, Found: 546.1835.

(*E*)-4-((4-Acetylphenyl)(4-fluorophenyl)methylene)-2-benzyl-3,4-dihydroisoquinolin-1(2*H*)-one (4fc): 70.0 mg (80%) as colourless solid; mp 139-140; AT-IR: 1679, 1645, 1599, 1505, 1261, 763, 628 cm^{-1}; ^{1}H NMR (400 MHz, $CDCl_3$) δ = 8.06 (dd, *J* = 7.8, 1.0 Hz, 1H), 7.77-7.75 (m, 2H), 7.24 (ddd, *J* = 7.8, 1.0, 0.5 Hz, 1H), 7.10-7.06 (m, 3H), 7.05-7.01 (m, 3H), 6.95-6.93 (m, 2H), 6.92-6.88 (m, 2H), 6.83-6.79 (m, 2H), 6.70 (dd, *J* = 7.9, 0.6 Hz, 1H), 4.57 (s, 2H), 3.97 (s, 2H), 2.53 (s, 3H) ppm; ^{13}C NMR (101 MHz, $CDCl_3$) δ = 196.4, 162.9, 161.4 (d, $^{1}J_{CF}$ = 248.7 Hz), 144.8, 139.0, 135.7, 135.6, 135.5 (d, $^{4}J_{CF}$ = 3.5 Hz), 135.3, 131.5 (d, $^{3}J_{CF}$ = 8.1 Hz), 129.7, 128.8, 128.7, 127.5, 127.32, 127.30, 127.23, 127.21 (d, $^{5}J_{CF}$ = 0.7 Hz), 126.9, 126.3, 114.6 (d, $^{2}J_{CF}$ = 21.5 Hz), 49.2, 49.1, 25.6 6 ppm; HRMS: *m/z* (MALDI-TOF) positive ion, calculated for $C_{31}H_{24}FKNO_2$: $[M+K]^+$ 500.1428, Found: 500.1435.

(*E*)-4-((4-Acetylphenyl)(4-methoxyphenyl)methylene)-2-benzyl-3,4-dihydroisoquinolin-1(2*H*)-one (4fj): 75.0 mg (92%); Colourless solid; mp 143-144; AT-IR: 3066, 3036, 1689, 1653, 1603, 1511, 1246, 768, 697 cm^{-1}; ^{1}H NMR (400 MHz, $CDCl_3$) δ = 8.05 (d, *J*= 7.8 Hz, 1H), 7.75 (d, *J* = 8.2 Hz, 2H), 7.22 (td, *J* = 7.7, 0.9 Hz, 1H), 7.09-7.03 (m, 6H), 6.96 (d, *J* = 8.2 Hz, 2H), 6.83 (d, *J* = 8.7 Hz, 2H), 6.77 (d, J = 7.8 Hz, 1H), 6.68-6.60 (m, 2H), 4.57 (s, 2H), 3.96 (s, 2H), 3.70 (s, 3H), 2.53 (s, 3H) ppm;^{13}C NMR (101 MHz, $CDCl_3$) δ = 197.4, 164.0, 159.4, 146.4, 140.8, 137.1, 136.9, 136.2, 132.8, 132.1, 130.6, 129.8, 128.5, 128.4, 128.2, 127.9, 127.3, 127.0, 113.9, 55.2, 50.3, 50.1, 26.6 ppm; HRMS: *m/z* (MALDI-TOF) positive ion, calculated for $C_{32}H_{27}KNO_3$: $[M+K]^+$ 512.1628, Found: 512.1628.

(*E*)-2-Benzyl-4-((4-methoxyphenyl)(2-phenyl-1H-indol-3-yl)methylene)-3,3-dimethyl-3,4-dihydroisoquinolin-1(2*H*)-one (6ba): 40.0 mg (70%) as pale yellow powder; mp 186-188 °C; AT-IR: 3279, 1630, 1599, 1507, 1248, 1029, 769, 744, 693 cm^{-1}; ^{1}H NMR (400 MHz, CDCl3) δ = 7.88 (s, 1H), 7.58 (dd, *J* = 7.7, 0.8 Hz, 1H), 7.37 (dd, *J* = 8.3, 2.1 Hz, 1H), 7.22 (dd, *J* = 6.7, 5.4 Hz, 2H), 7.20-7.10 (m, 8H), 7.06-6.99 (m, 4H), 6.97-6.91 (m, 1H), 6.88 (t, *J* = 7.5 Hz, 1H), 6.80 (td, *J* = 8.3, 2.0 Hz, 2H), 6.73 (d, *J* = 7.6 Hz, 1H), 6.66 (dd, *J* = 8.5, 2.7 Hz, 1H), 4.86 (d, *J* = 16.2 Hz, 1H), 4.47 (d, *J* = 16.2 Hz, 1H), 3.73 (s, 3H), 1.20 (s, 3H), 1.12 (s, 3H) ppm; ^{13}C NMR (101 MHz, $CDCl_3$) δ = 165.1, 159.0, 140.3, 140.1, 137.0, 136.8, 136.3, 135.9, 133.4, 132.1, 131.8, 130.6, 129.9, 129.8, 129.3, 128.7, 128.4, 128.3, 128.1, 127.6, 127.3, 126.8, 126.5, 126.22, 126.18, 122.1, 120.4, 113.9, 112.7, 110.8, 63.4, 55.2, 45.8, 29.7, 28.3 ppm; HRMS: *m/z* (MALDI-TOF) positive ion, calculated for $C_{40}H_{34}N_2O_2$: $[M]^+$ 574.2620, Found: 574.2614.

(*E*)-4-((4-Acetylphenyl)(2-phenyl-1H-indol-3-yl)methylene)-2-benzyl-3,4-dihydroisoquinolin-1(2*H*)-one (6fa): 49.0 mg (89%) as yellow powder; mp 210-211 °C; AT-IR: 3243, 3070, 1680, 1630, 1599, 1268, 741,698 cm^{-1}; ^{1}H NMR (400 MHz, CDCl3) δ = 8.25 (s, 1H), 7.76-7.73 (m, 2H), 7.67 (dd, *J* = 7.8, 1.0 Hz, 1H), 7.28 (d, *J* = 8.1 Hz, 1H), 7.23-7.16 (m, 2H), 7.11-6.96 (m, 12H), 6.90-6.82 (m, 3H), 6.75 (dd, *J* = 7.9, 0.7 Hz, 1H), 4.76 (d, *J* = 14.7 Hz, 1H, AB system), 4.26 (d, *J* = 14.7 Hz, 1H), 4.16 (d, *J* = 13.1 Hz, 1H, AB system), 4.10 (d, *J* = 13.1 Hz,

1H, AB system), 2.52 (s, 3H) ppm; ^{13}C NMR (101 MHz, $CDCl_3$) δ = 197.5, 163.3, 145.8, 138.2, 137.1, 136.9, 136.2, 136.0, 133.8, 131.7, 130.3, 129.7, 129.1, 129.0, 128.8, 128.41, 128.39, 128.17, 128.16, 128.1, 127.7, 127.29, 127.27, 126.8, 126.4, 122.6, 120.5, 119.8, 112.5, 111.0, 49.9, 49.7, 26.6 ppm; HRMS: *m/z* (MALDI-TOF) positive ion, calculated for $C_{39}H_{30}N_2NaO_2$: $[M+Na]^+$ 581.2205, Found: 581.2207.

(*E*)-4-((4-Acetylphenyl)(2-(thiophen-2-yl)-1*H*-indol-3-yl)methylene)-2-benzyl-3,4-dihydroisoquinolin-1(2*H*)-one (6fb): 49.0 mg (83%) as yellow solid; mp 159-160 °C;

AT-IR: 3217, 1682, 1631, 1599, 1265, 734 cm^{-1}; ^{1}H NMR (400 MHz, $CDCl_3$) δ = 8.12 (s, 1H), 7.81 (dd, *J* = 7.8, 1.0 Hz, 1H), 7.76 (d, *J* = 8.5 Hz, 2H), 7.25 (d, *J* = 8.1 Hz, 1H), 7.20-7.15 (m, 3H), 7.11-7.02 (m, 7H), 6.99-6.94 (m, 1H), 6.91-6.83 (m, 2H), 6.78 – 6.72 (m, 2H), 6.69 (dd, *J* = 3.6, 1.2 Hz, 1H), 4.87 (d, J = 14.4 Hz, 1H), 4.23 (d, J = 14.4 Hz, 1H), 4.21 (s, 2H), 2.53 (s, 3H) ppm; ^{13}C NMR (101 MHz, $CDCl_3$) δ = 197.6, 163.5, 145.6, 137.7, 137.0, 136.2, 136.0, 133.3, 132.9, 130.5, 130.3, 130.00, 129.6, 129.2, 129.0, 128.5, 128.4, 128.2, 127.75, 127.71, 127.4, 127.3, 126.3, 126.1, 124.9, 123.1, 120.8, 119.6, 113.3, 110.9, 50.0, 49.7, 26.6 ppm; HRMS: *m/z* (ESI) positive ion, calculated for $C_{37}H_{29}N_2O_2S$: $[M+H]^+$ 565.1950, Found: 565.1952.

(*E*)-4-((4-Acetylphenyl)(2-((*E*)-cyclooct-1-en-1-yl)-1*H*-indol-3-yl)methylene)-2-benzyl-3,4-dihydroisoquinolin-1(2*H*)-one (6fc): 47.0 mg (79%) as orange solid; mp 155-156 °C; AT-IR: 3523, 3242, 1674, 1633, 1600, 1266, 732, 697 cm^{-1}; ^{1}H NMR (400 MHz, $CDCl_3$) δ = 7.98 (dd, *J* = 7.9, 1.0 Hz, 1H), 7.86 (s, 1H) 7.84 (dd, *J* = 8.6, 1.8 Hz,1H), 7.74 (d, *J* = 8.5 Hz, 1H), 7.21-7.16 (m, 2H), 7.13-7.06 (m, 3H), 7.05-6.96 (m, 5H), 6.89 (td, *J* = 7.6, 1.4 Hz, 1H), 6.78 (d, *J* = 0.7 Hz, 2H), 6.73 (dd, *J* = 7.6, 0.7 Hz, 1H), 5.55 (t, *J* = 8.2 Hz, 1H), 4.82 (d, *J* = 14.6 Hz, 1H), 4.36 (d, J = 14.6 Hz, 1H), 4.18 (s, 2H), 2.53 (s, 3H), 2.23-2.08 (m, 1H), 2.04-1.93 (m, 1H), 1.89-1.77 (m, 2H), 1.49 (d, *J* = 5.9 Hz, 2H), 1.41-1.14 (m, 6H) ppm; ^{13}C NMR (101 MHz, $CDCl_3$) δ = 197.5, 163.9, 145.8, 139.1, 138.7, 137.0, 136.2, 135.0, 134.7, 132.7, 131.4, 130.6, 129.8, 129.3, 128.9, 128.7, 128.4, 128.2, 128.0, 127.8, 127.3, 127.2, 126.7, 122.0, 120.2, 119.3, 111.6, 110.5, 50.0, 49.7, 31.6, 29.4, 27.3, 26.8, 26.6, 26.5, 25.7 ppm; HRMS: *m/z* (MALDI-TOF) positive ion, calculated for $C_{41}H_{38}N_2O_2$: $[M]^+$ 590.2933, Found: 590.2935.

1-(4-(3-(Benzylamino)prop-1-yn-1-yl)phenyl)ethenone (1f)

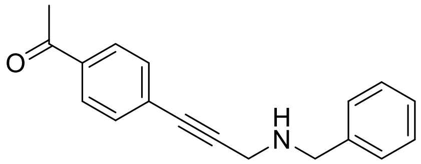

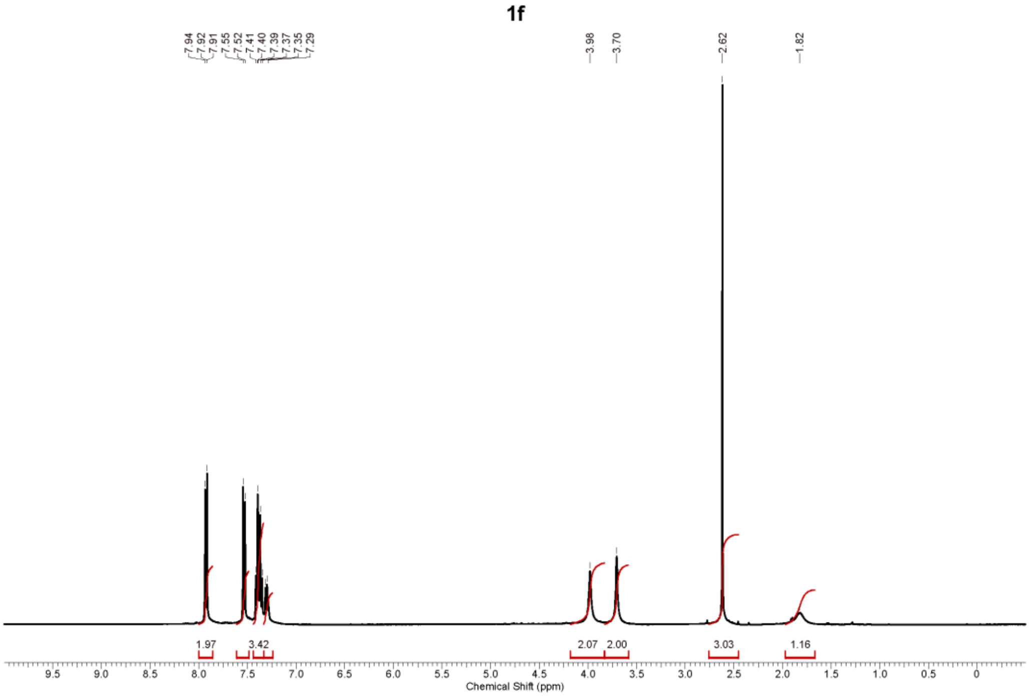

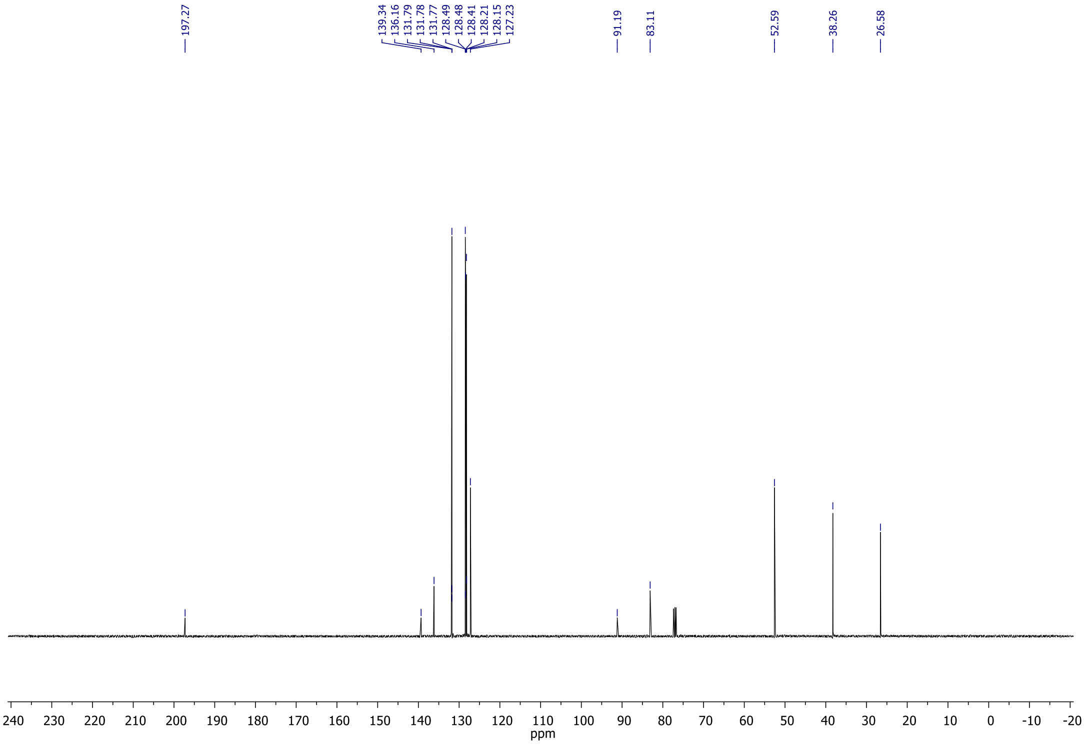

***N*-(4-(4-Acetylphenyl)-2-methylbut-3-yn-2-yl)-*N*-benzyl-2-iodobenzamide (2a)**

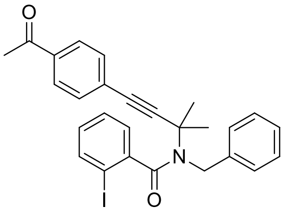

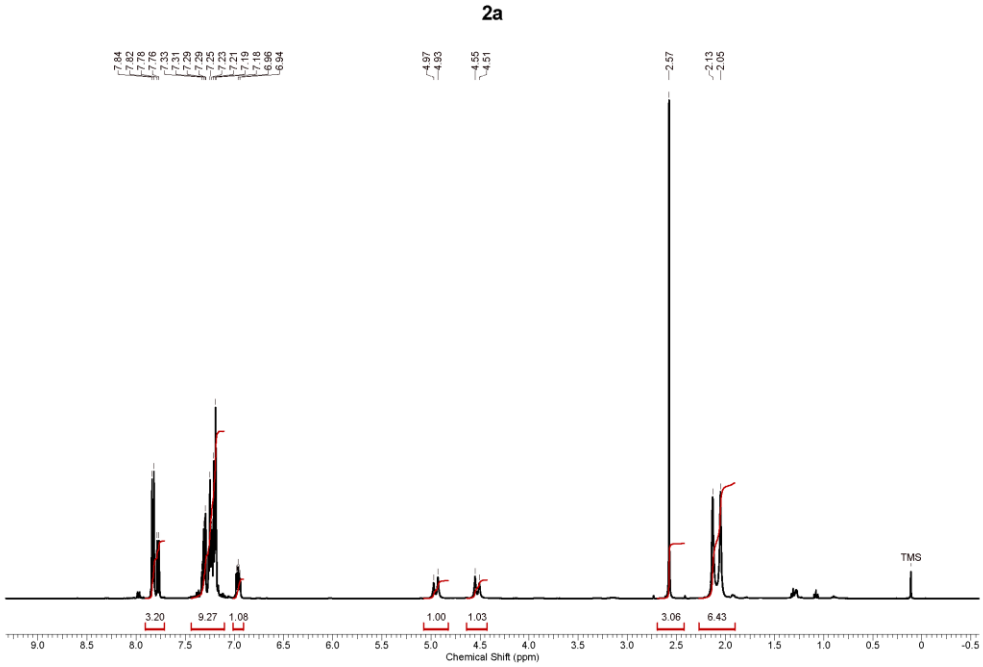

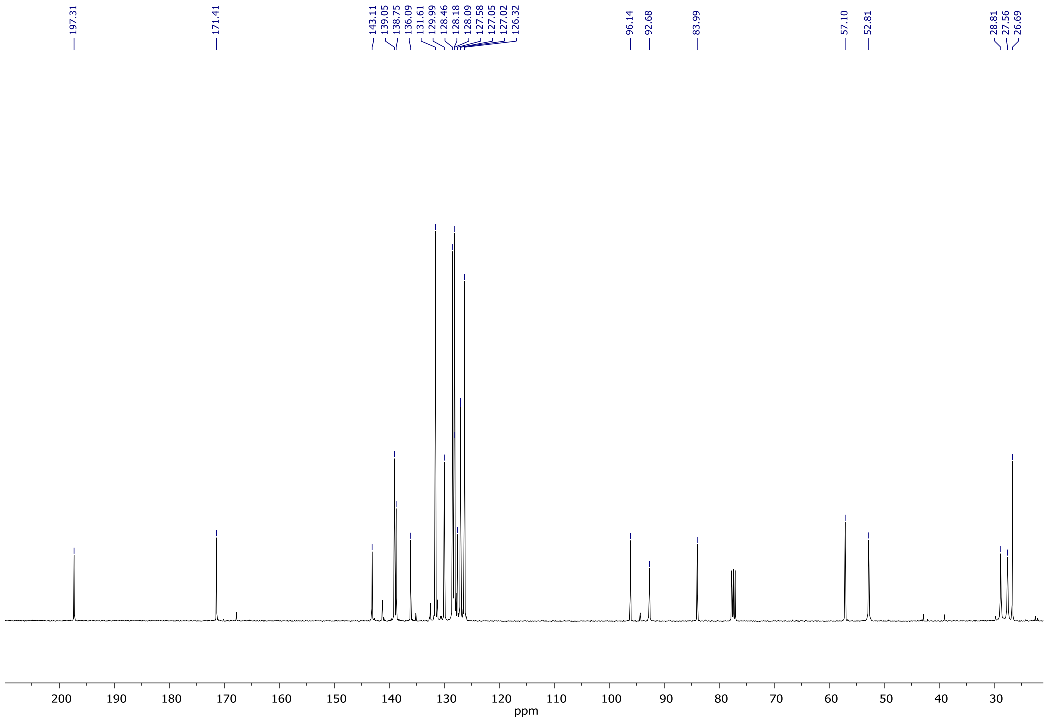

***N*-Benzyl-2-iodo-*N*-(4-(4-methoxyphenyl)-2-methylbut-3-yn-2-yl)benzamide (2b)**

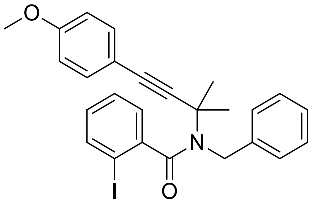

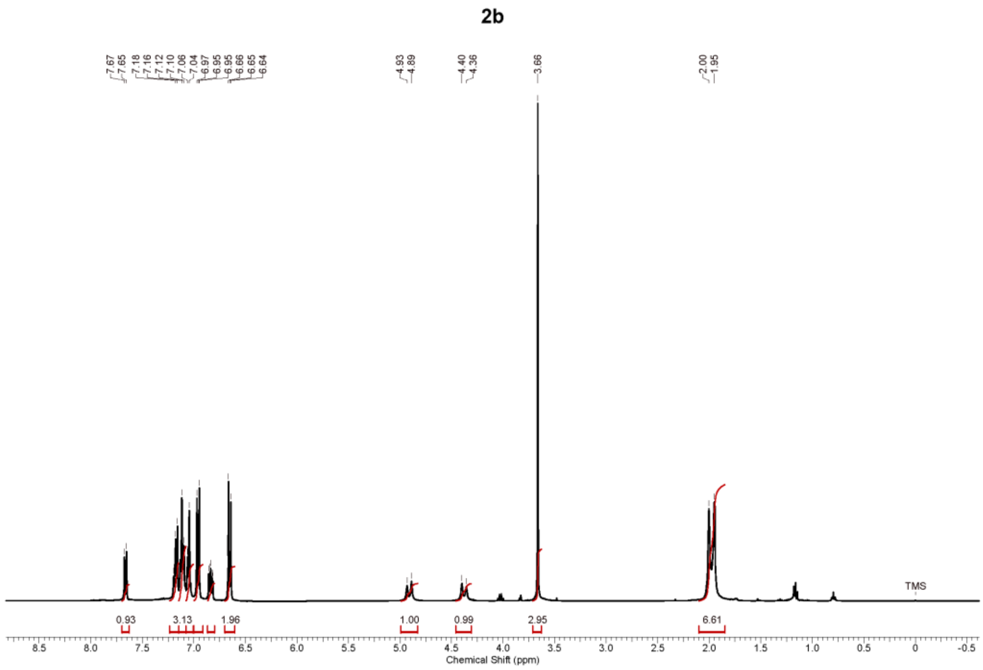

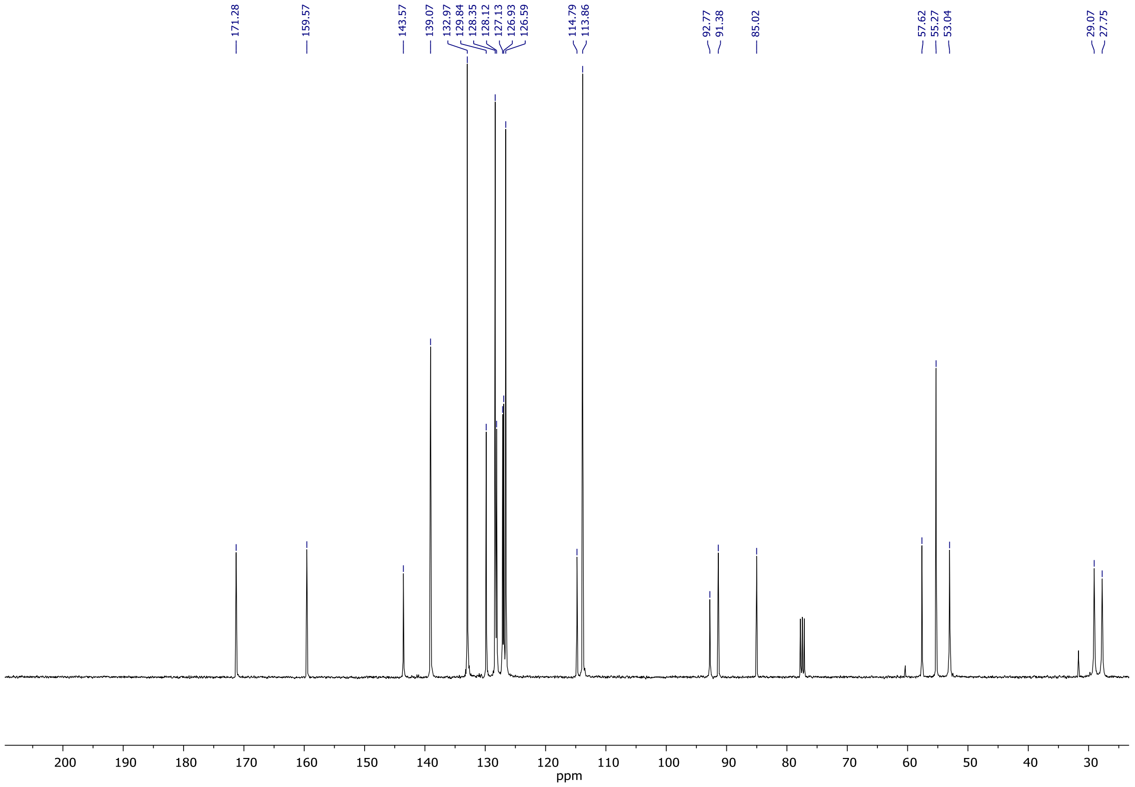

_N_-Benzyl-2-iodo-_N_-(2-methyl-4-(4-(trifluoromethyl)phenyl)but-3-yn-2-yl)benzamide (2c)

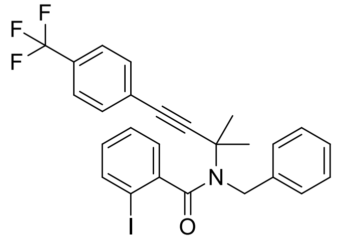

2c

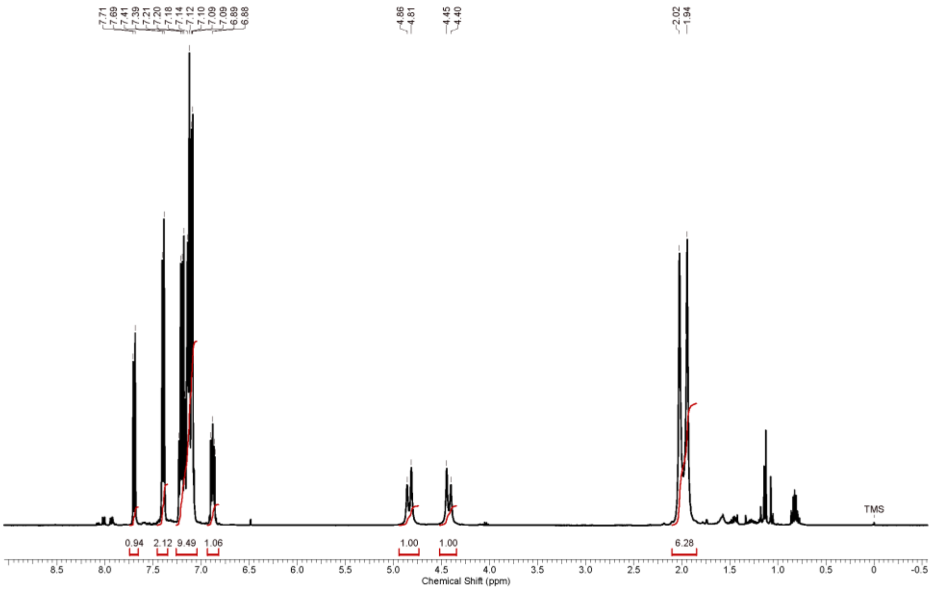

2c

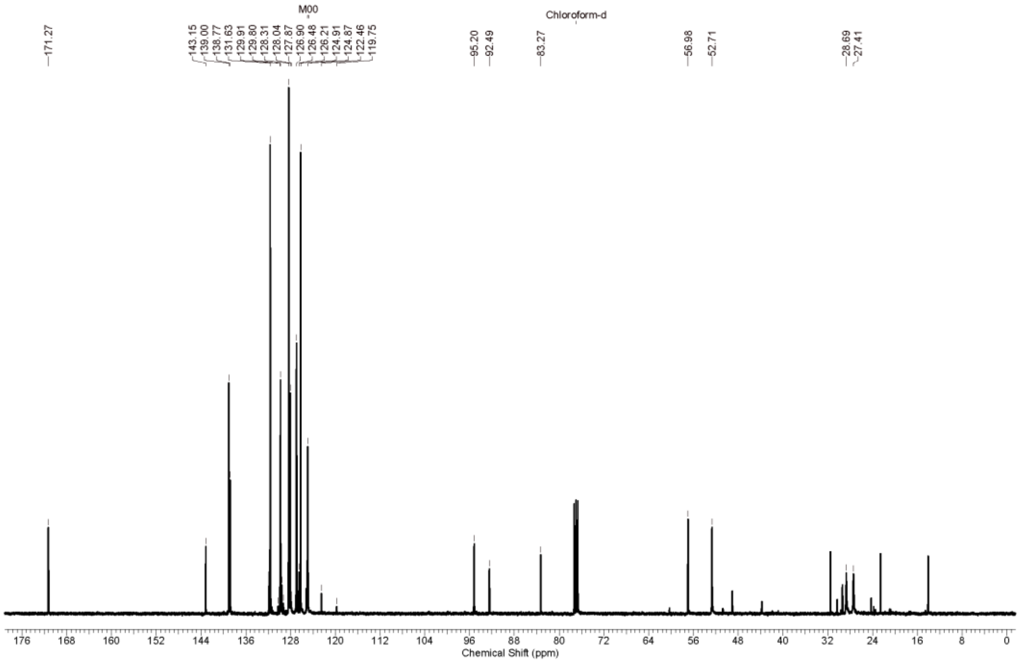

_N_-Benzyl-2-iodo-_N_-(1-(oct-1-yn-1-yl)cyclohexyl)benzamide (2d)

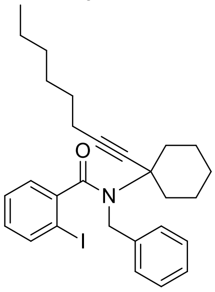

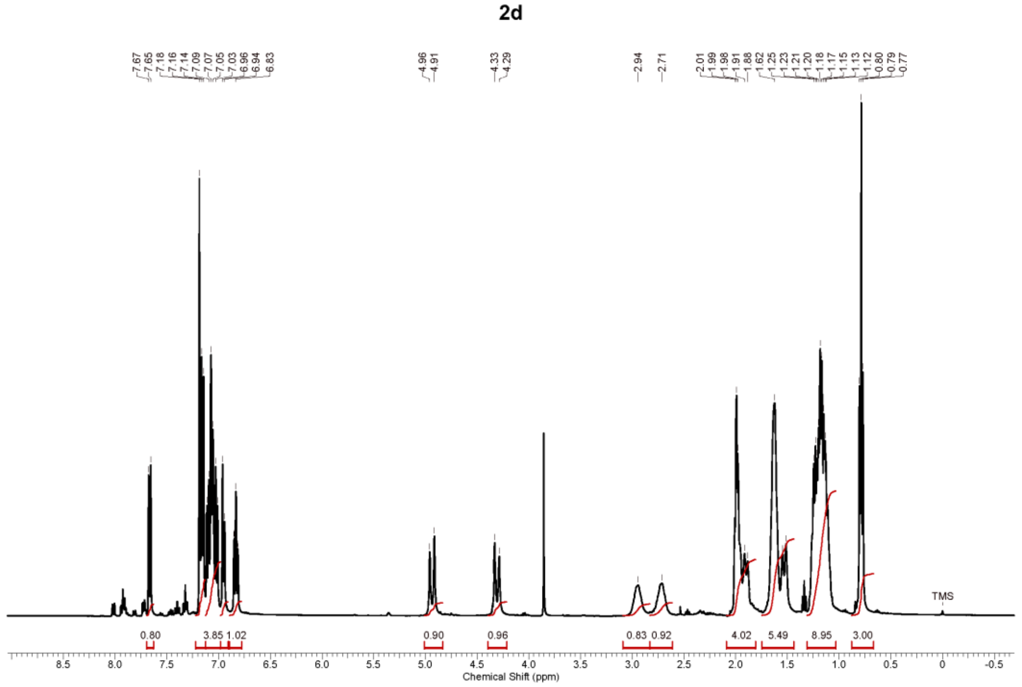

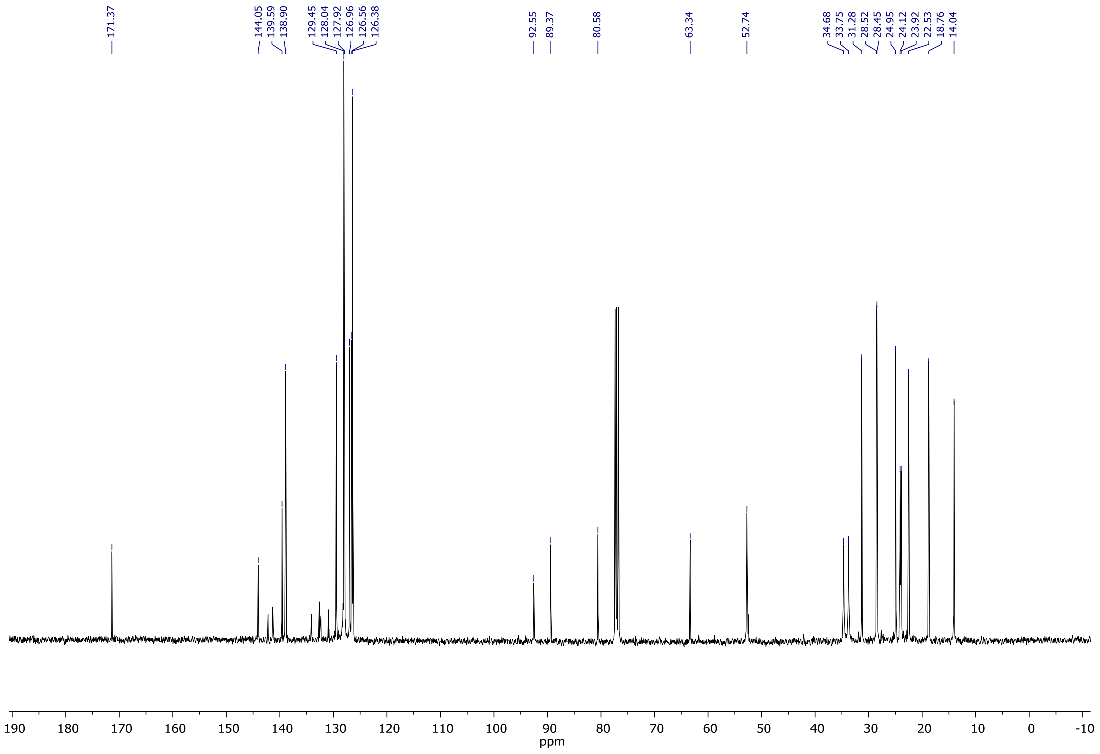

***N*-Benzyl-*N*-(1,3-diphenylprop-2-yn-1-yl)-2-iodobenzamide (complex mixture of slowly interconverting amide geometric (1*S*,1*E*/1*S*,1*Z*) and (1*R*,1*E*/1*R*,1*Z*) isomers) (2e)**

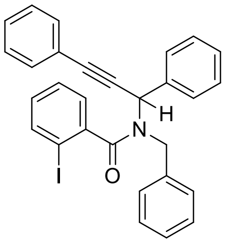

spectrum at 303 K

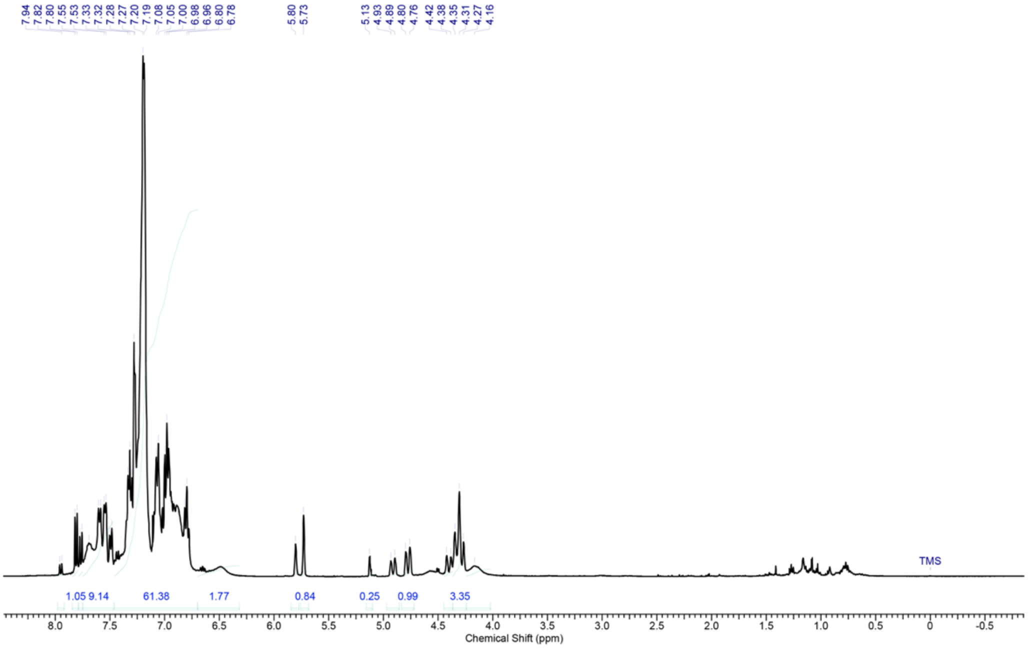

spectrum at 323 K

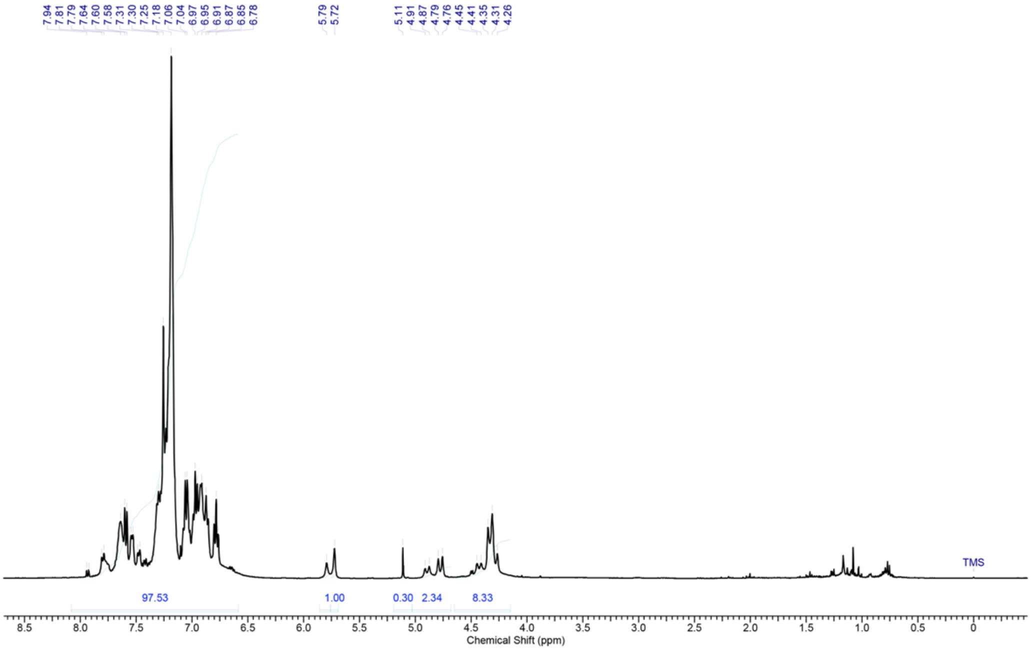

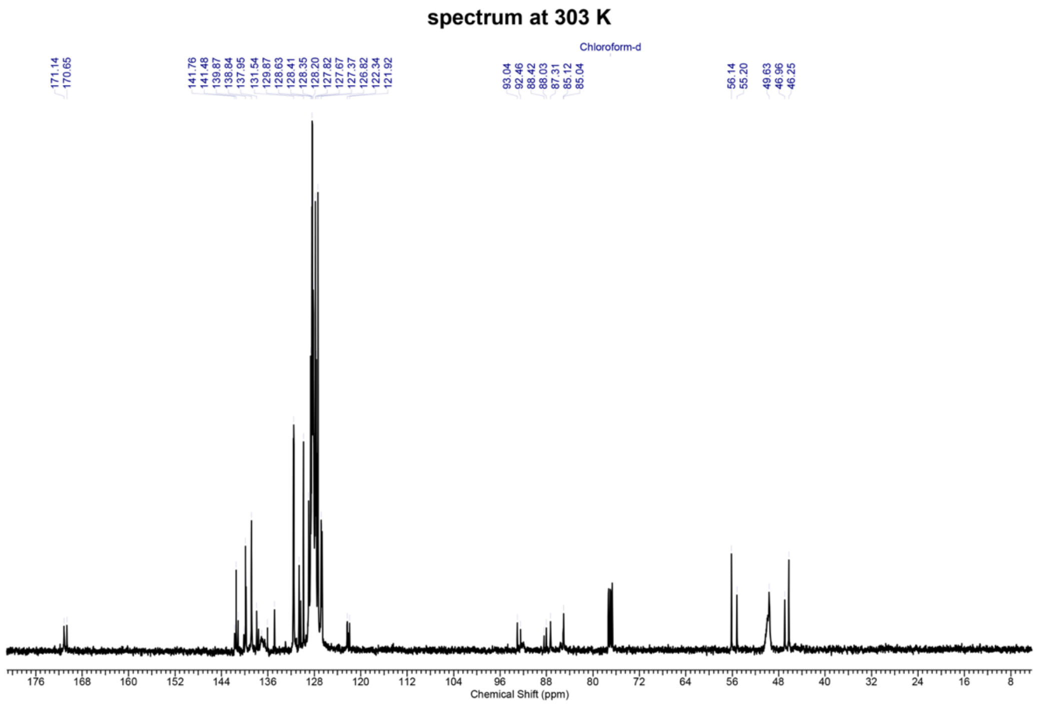
spectrum at 303 K
Chloroform-d
171.14
170.65
141.76
141.48
139.87
138.84
137.95
131.54
129.87
128.63
128.41
128.35
128.20
127.82
127.67
127.37
126.82
122.34
121.92
93.04
92.46
88.42
88.03
87.31
85.12
85.04
56.14
55.20
49.63
46.96
46.25
Chemical Shift (ppm)

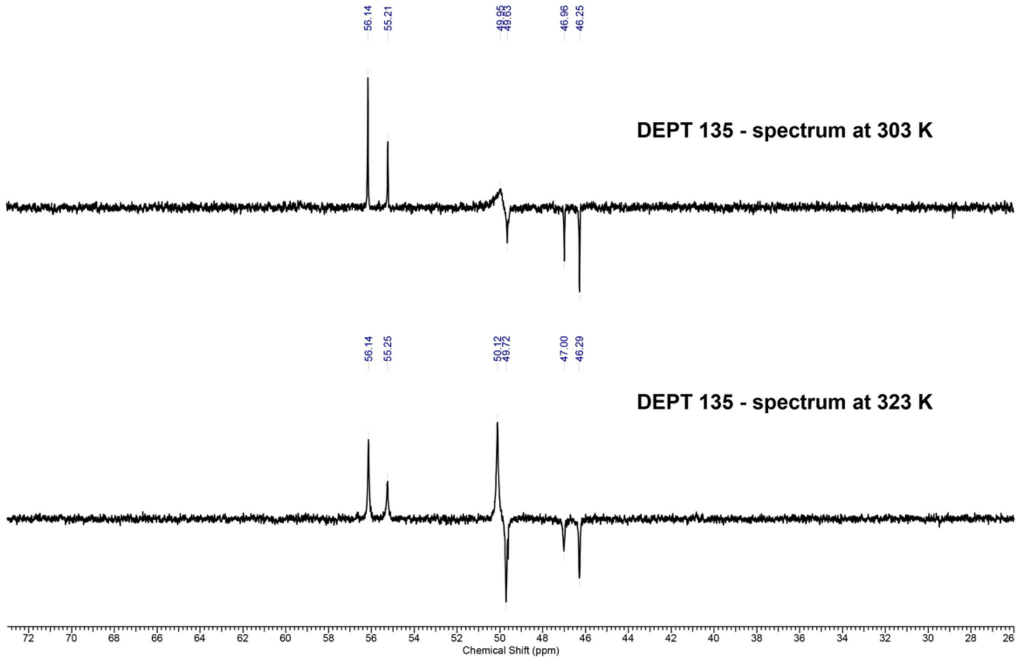
56.14
55.21
49.95
49.63
46.96
46.25
DEPT 135 - spectrum at 303 K
56.14
55.25
50.12
49.72
47.00
46.29
DEPT 135 - spectrum at 323 K
Chemical Shift (ppm)

N-(3-(4-acetylphenyl)prop-2-yn-1-yl)-*N*-benzyl-2-iodobenzamide (mixture of slowly interconverting geometric *E*/*Z* isomers) (2f)

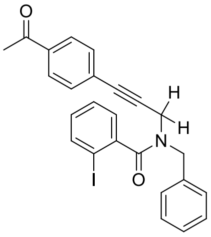

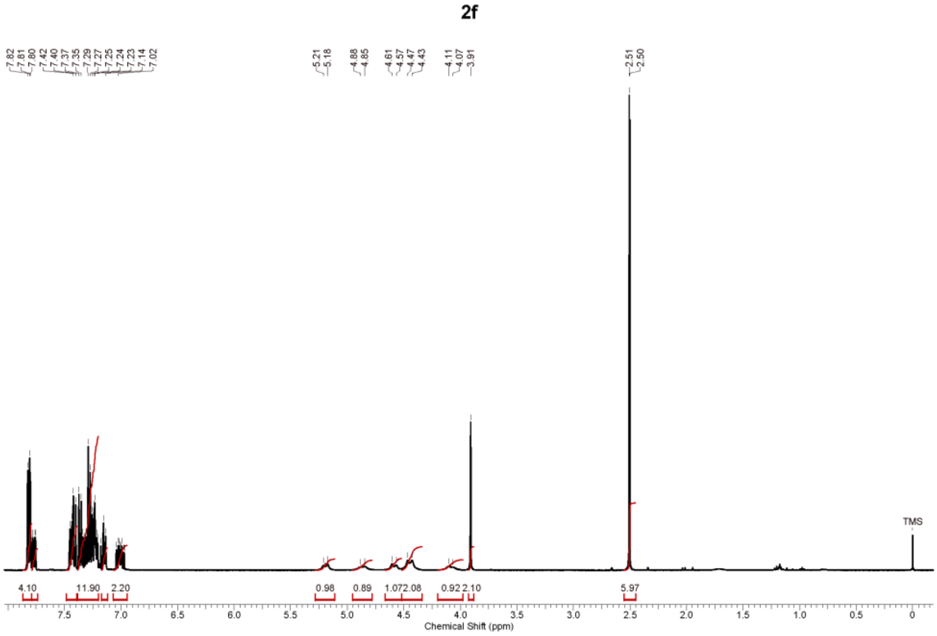

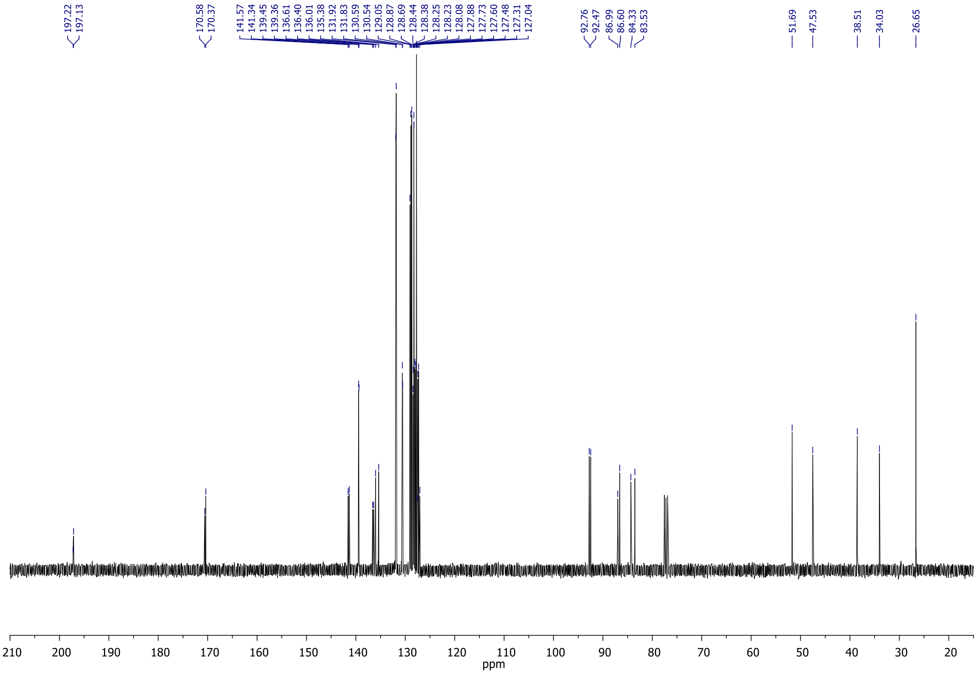

(*Z*)-4-((4-Acetylphenyl)(phenyl)methylene)-2-benzyl-3,3-dimethyl-3,4-dihydroisoquinolin-1(2*H*)-one (4aa)

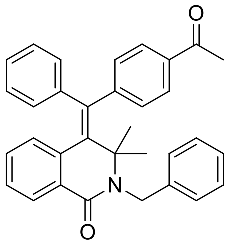

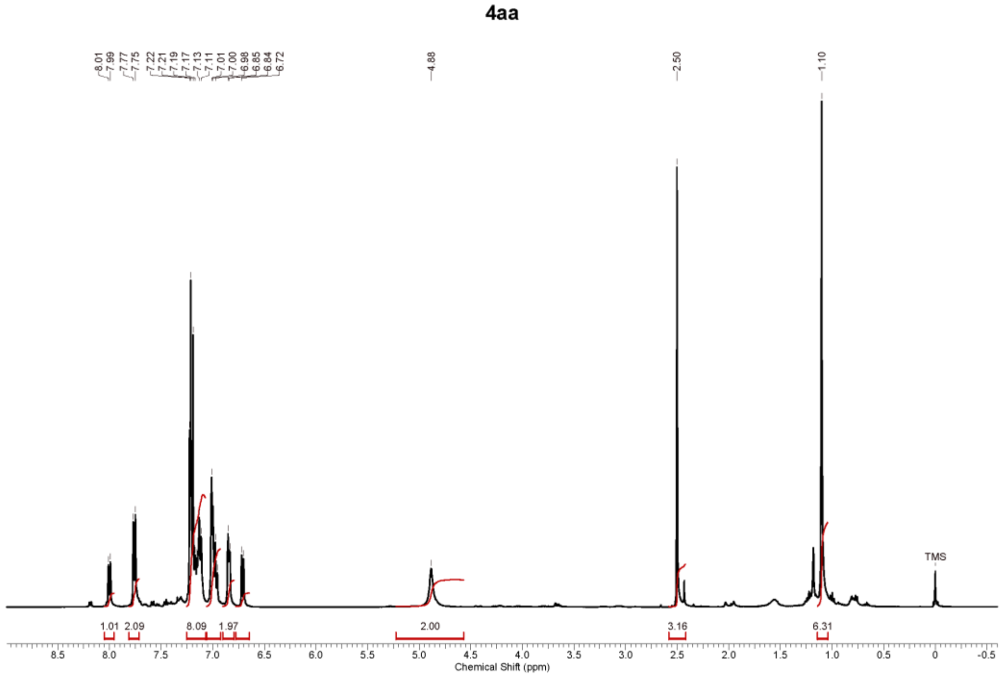

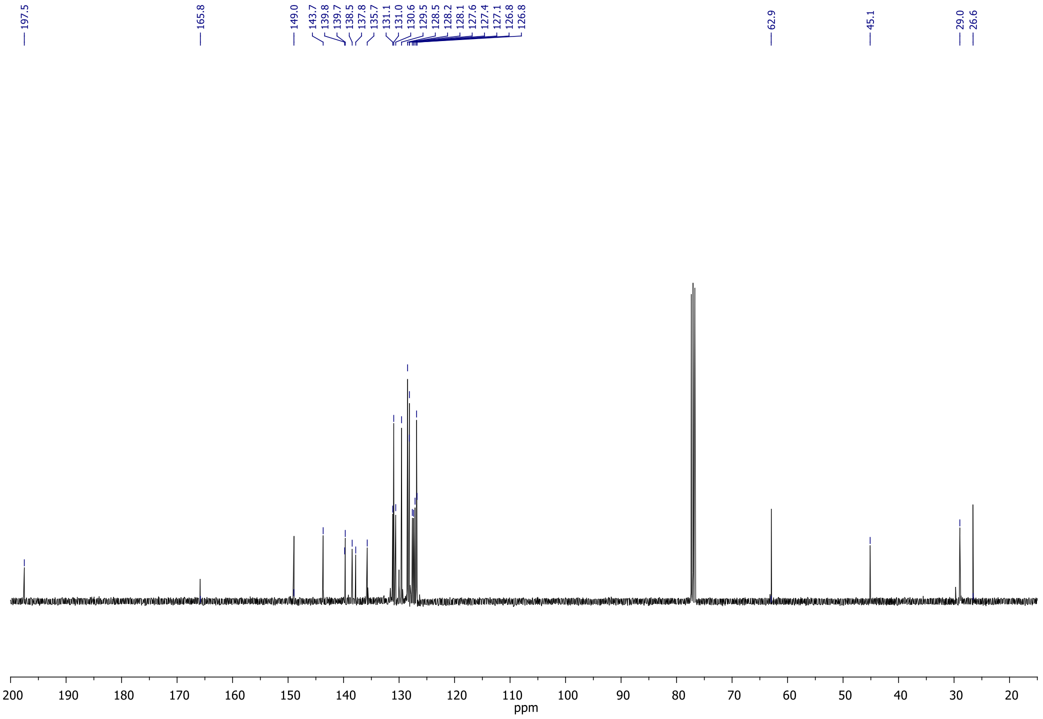

(*E*)-4-((4-Acetylphenyl)(3-methoxyphenyl)methylene)-2-benzyl-3,3-dimethyl-3,4-dihydroisoquinolin-1(2*H*)-one (4ab)

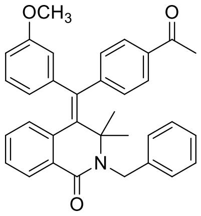

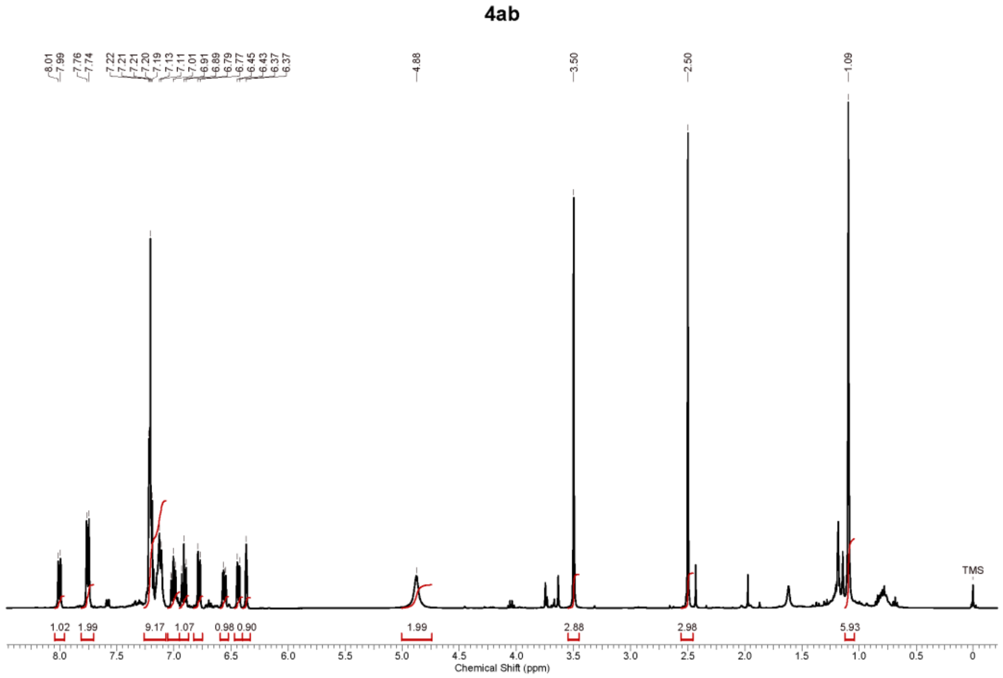

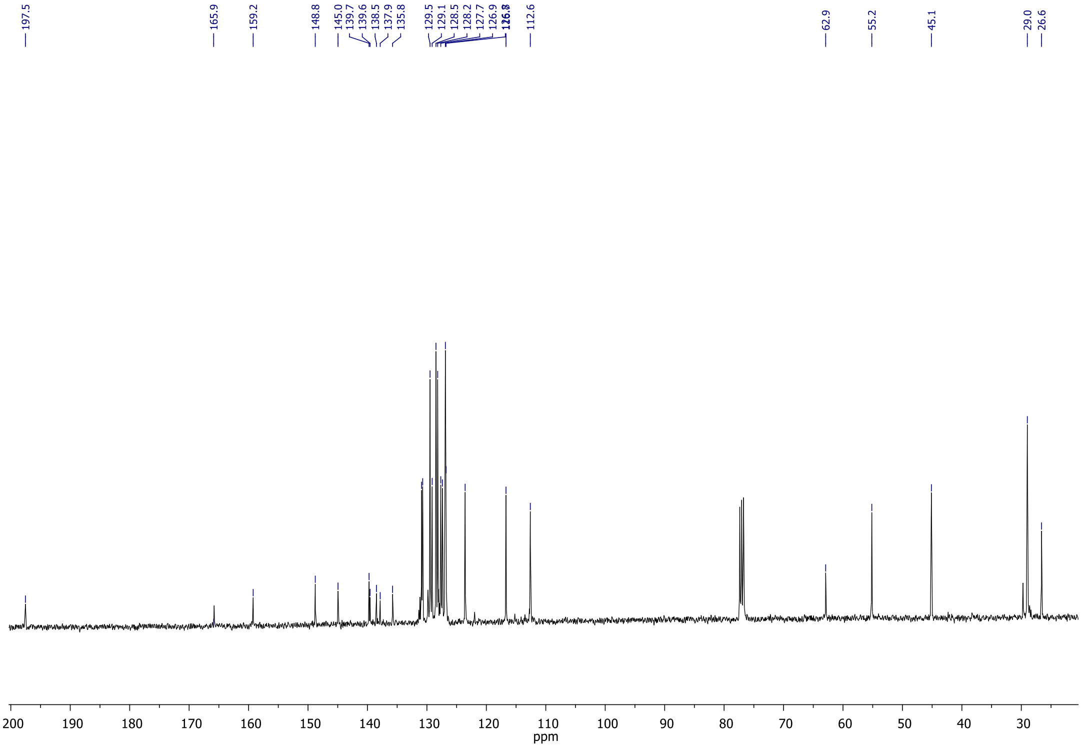

(*E*)-4-((4-Acetylphenyl)(4-fluorophenyl)methylene)-2-benzyl-3,3-dimethyl-3,4-dihydroisoquinolin-1(2*H*)-one (4ac)

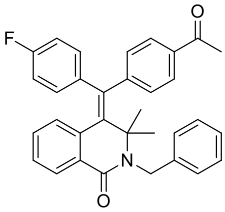

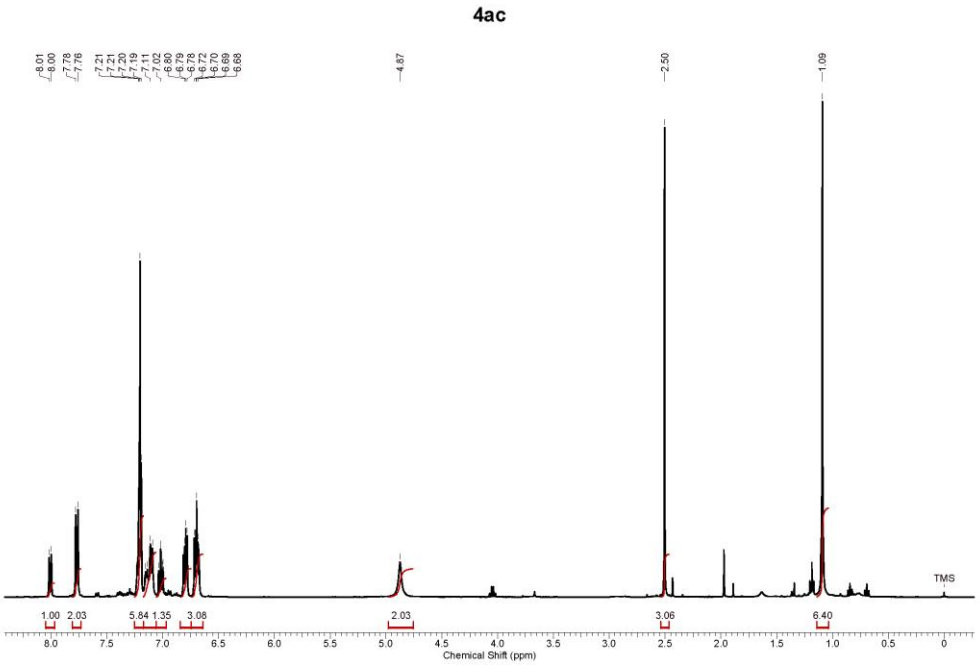

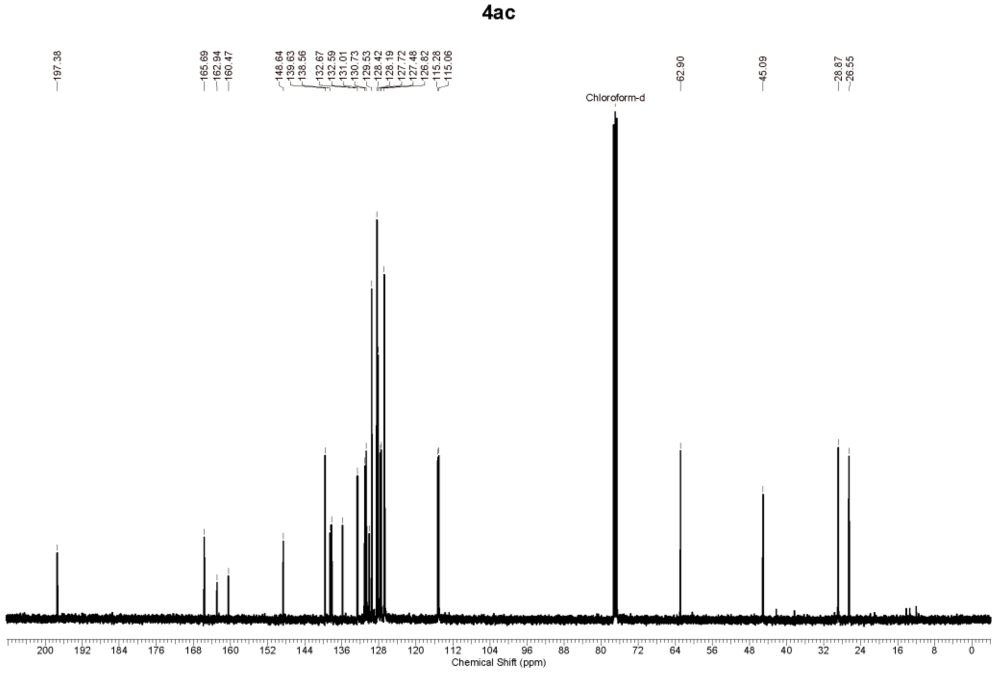

(*E*)-N-(3-((4-Acetylphenyl)(2-benzyl-3,3-dimethyl-1-oxo-2,3-dihydroisoquinolin-4(1*H*)ylidene)methyl)phenyl)acetamide (4ad)

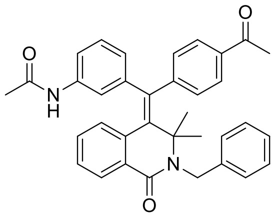

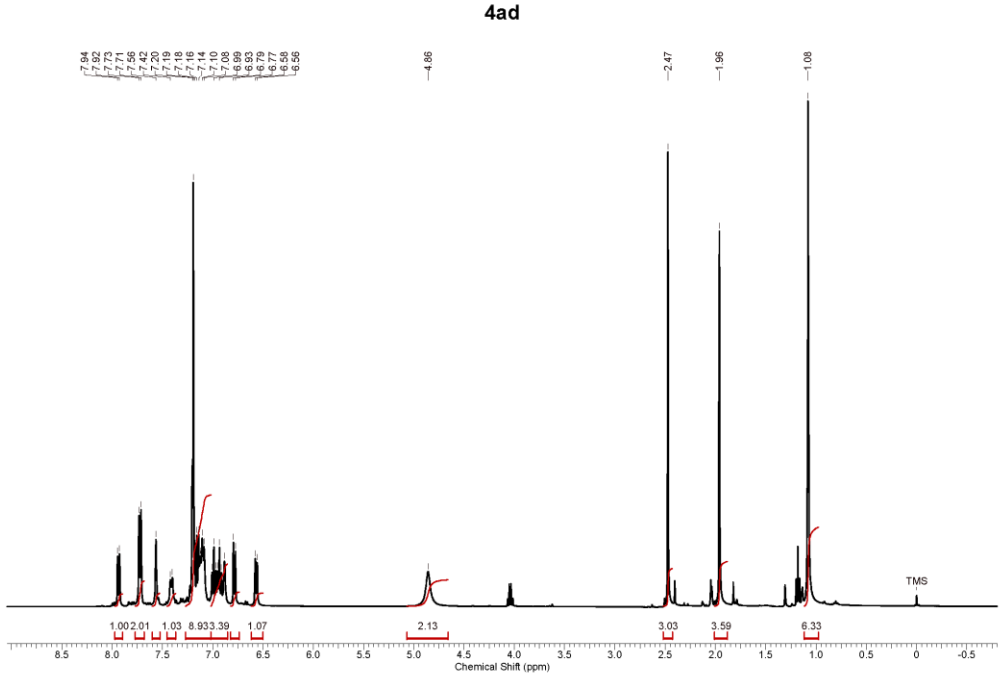

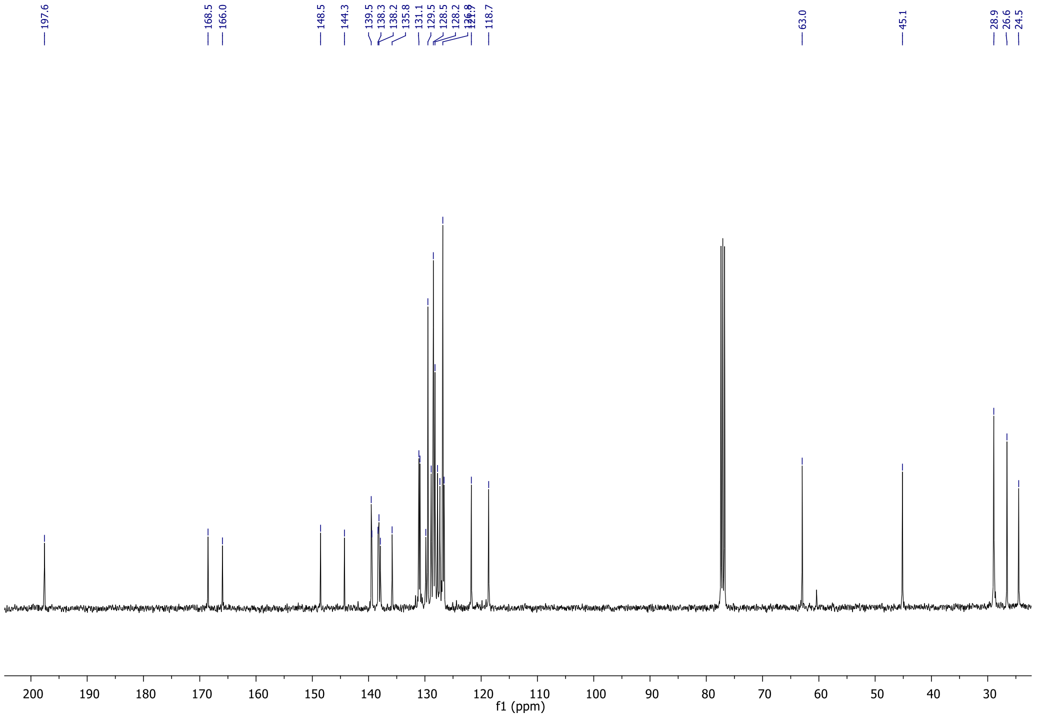

(*E*)-4-((4-Acetylphenyl)(4-(dimethylamino)phenyl)methylene)-2-benzyl-3,3-dimethyl-3,4-dihydroisoquinolin-1(2*H*)-one (4ae)

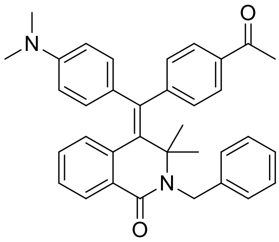

4ae

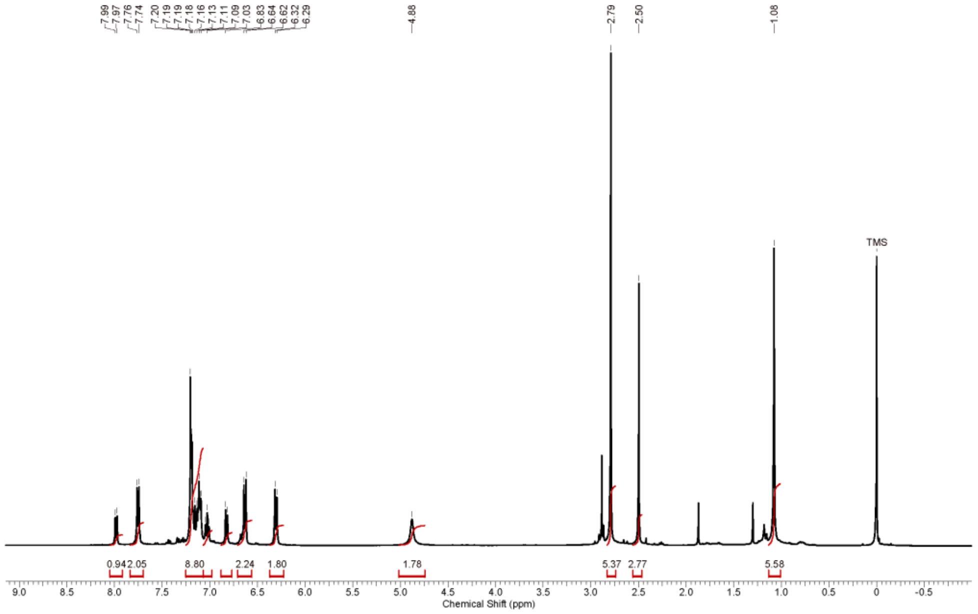

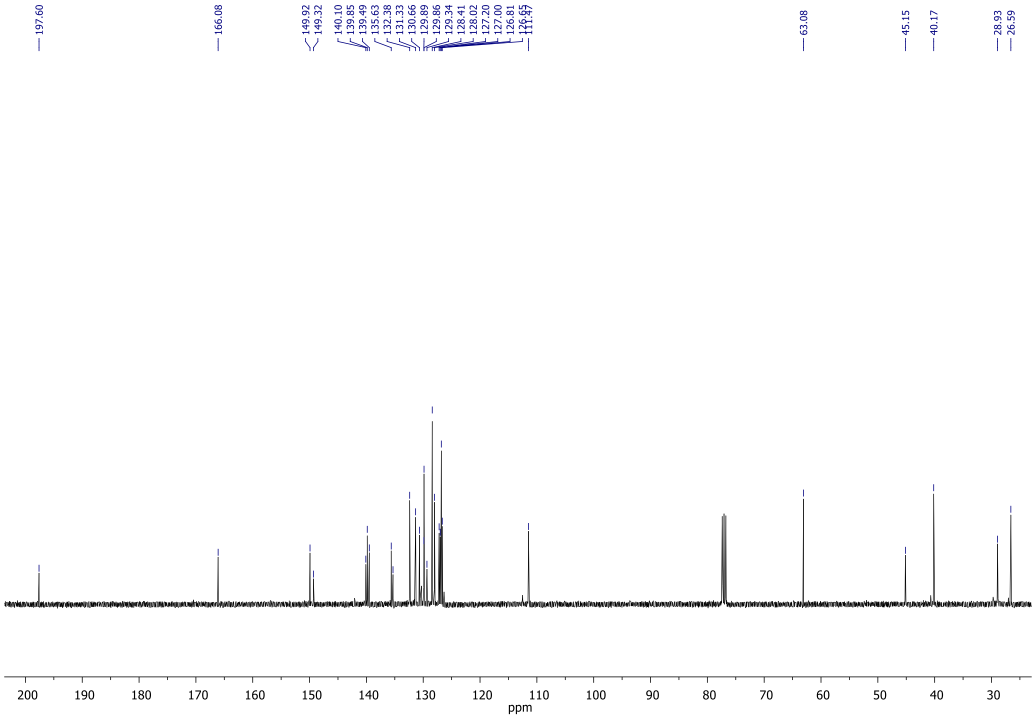

(*E*)-4-((4-Acetylphenyl)(3,5-dimethylphenyl)methylene)-2-benzyl-3,3-dimethyl-3,4-dihydroisoquinolin-1(2*H*)-one (4af)

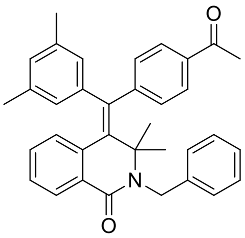

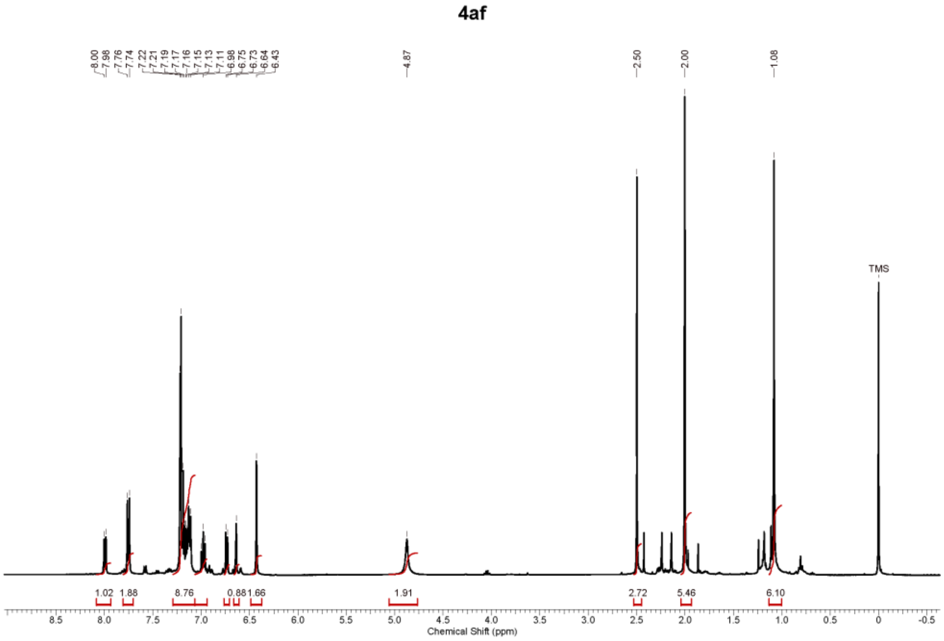

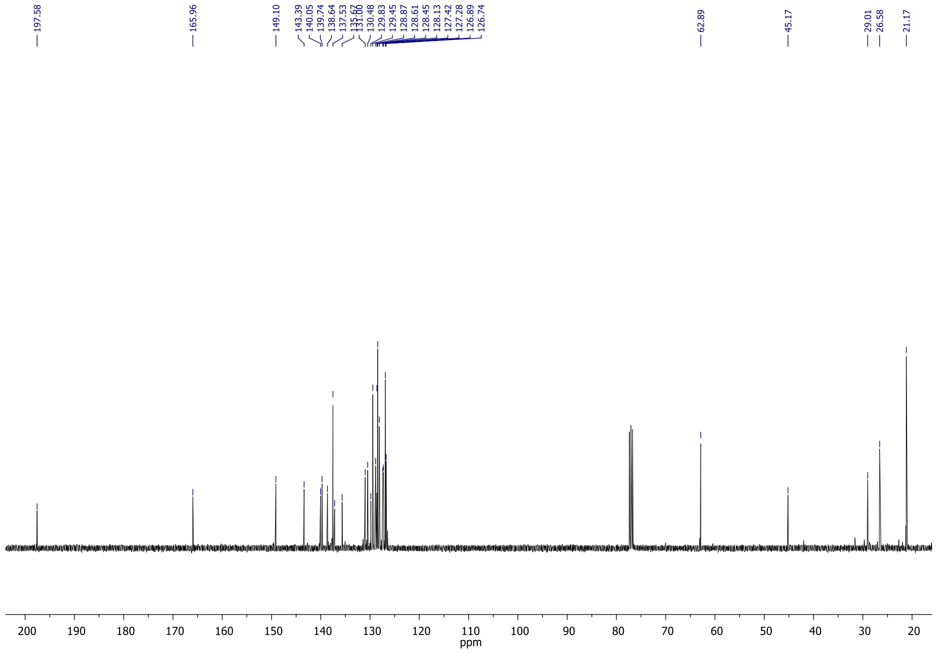

(*E*)-3-((4-Acetylphenyl)(2-benzyl-3,3-dimethyl-1-oxo-2,3-dihydroisoquinolin-4(1*H*)-ylidene)methyl)benzonitrile (4ag)

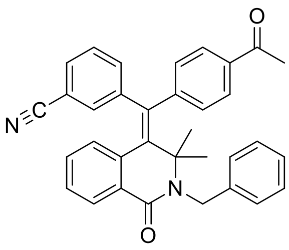

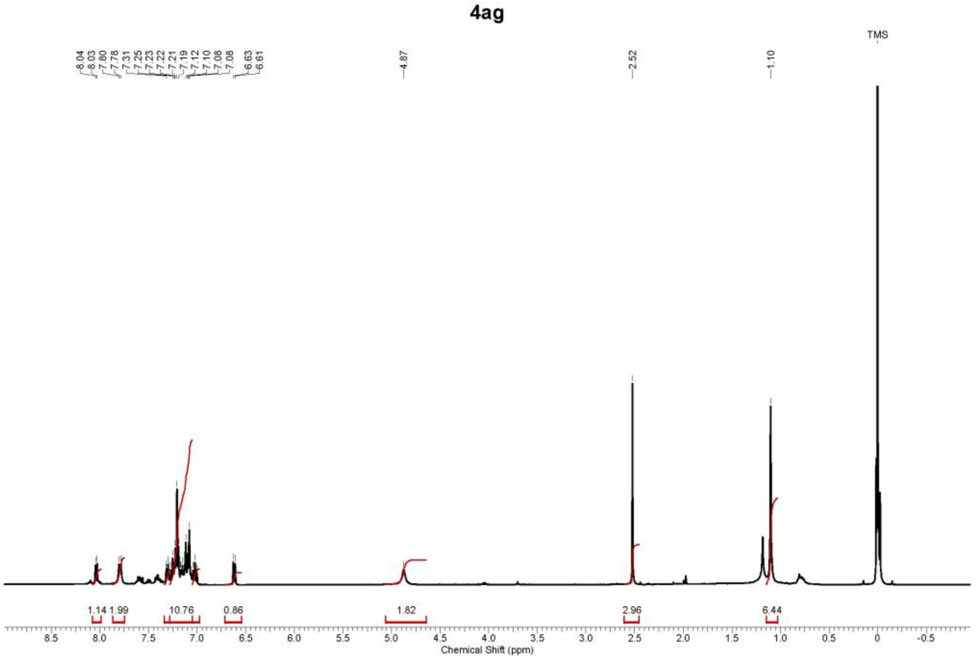

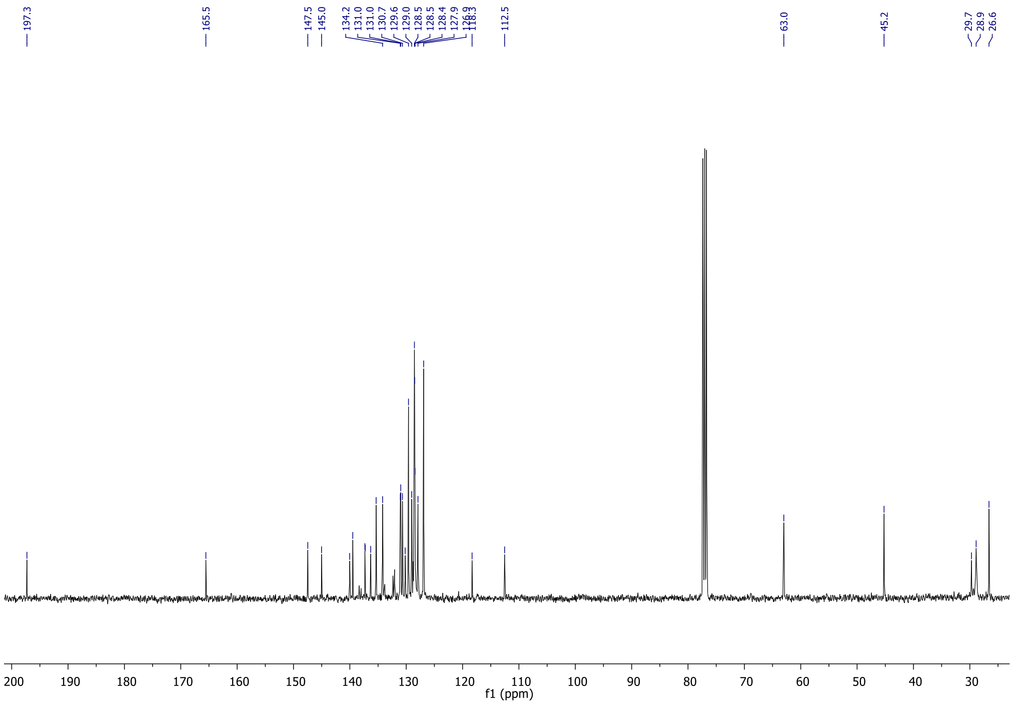

(*E*)-4-((4-Acetylphenyl)(3-(trifluoromethyl)phenyl)methylene)-2-benzyl-3,3-dimethyl-3,4-dihydroisoquinolin-1(2*H*)-one (4ah)

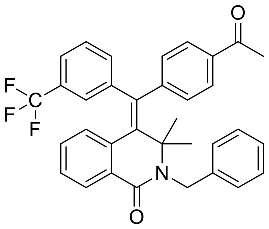

4ah

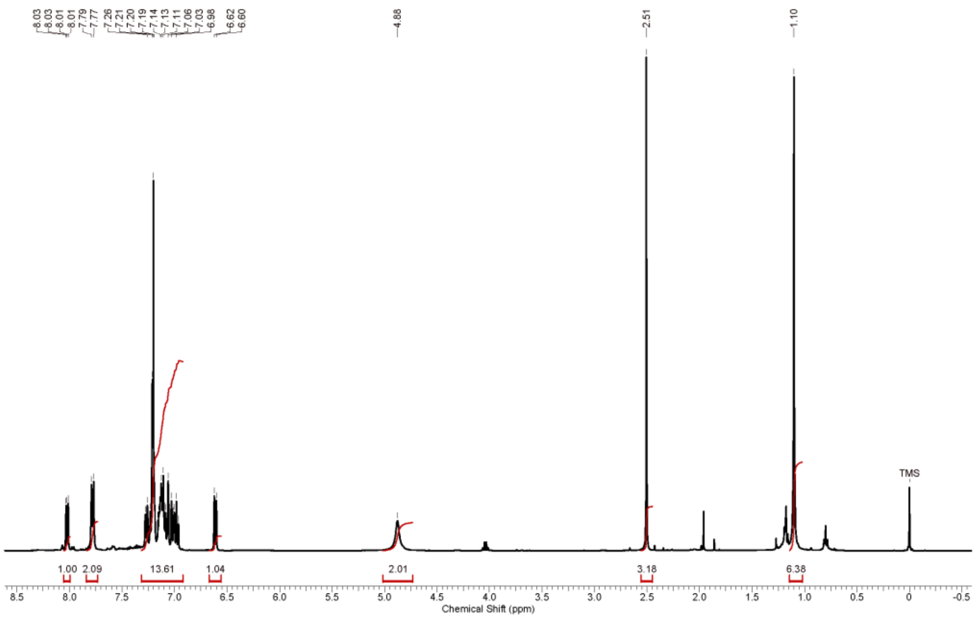

4ah

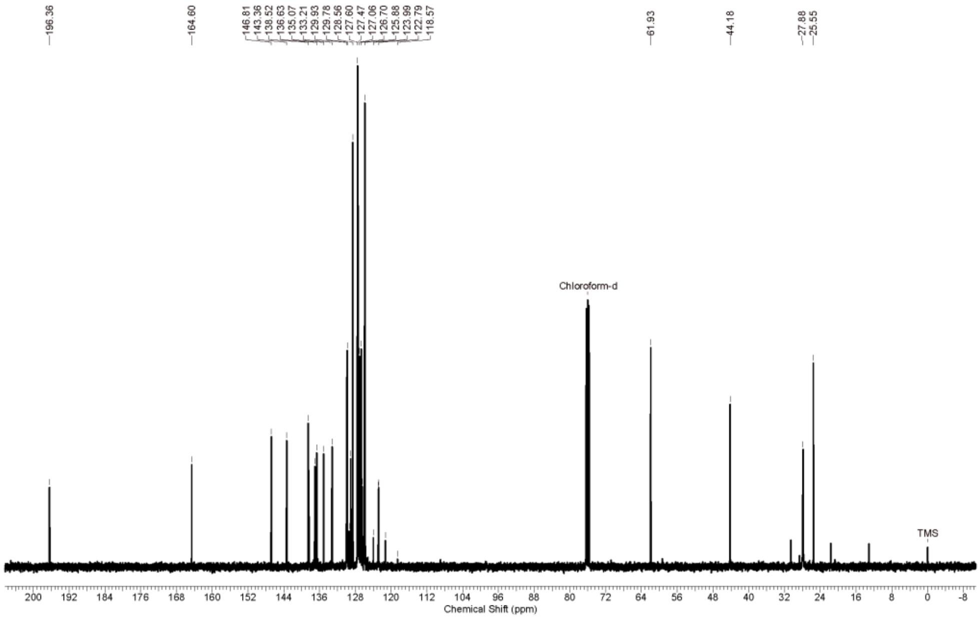

(*E*)-4-((4-Acetylphenyl)(3-chloro-4-fluorophenyl)methylene)-2-benzyl-3,3-dimethyl-3,4-dihydroisoquinolin-1(2*H*)-one (4ai)

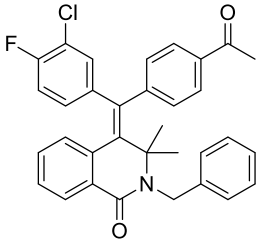

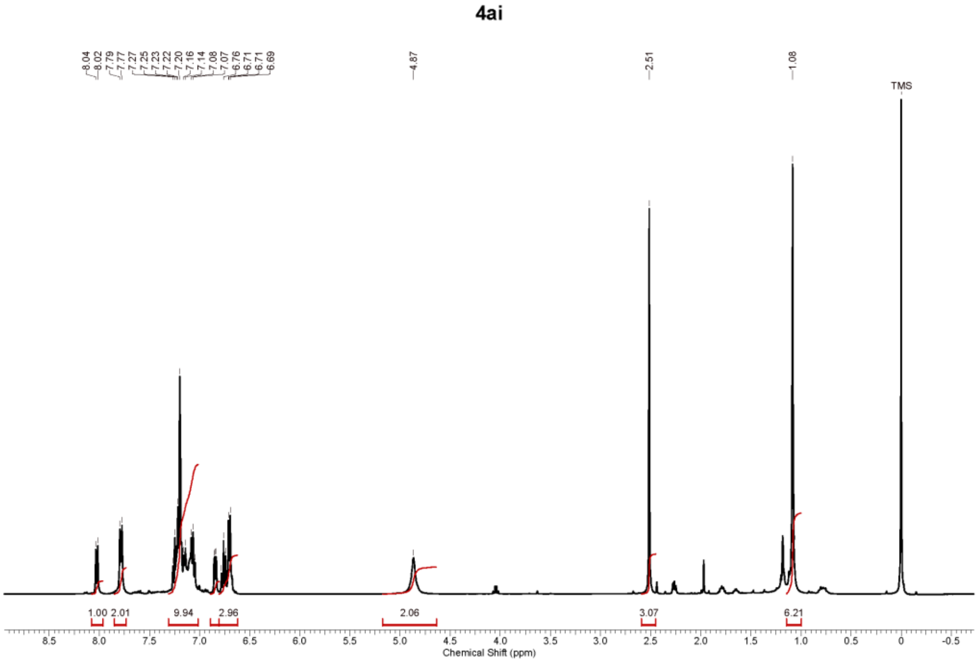

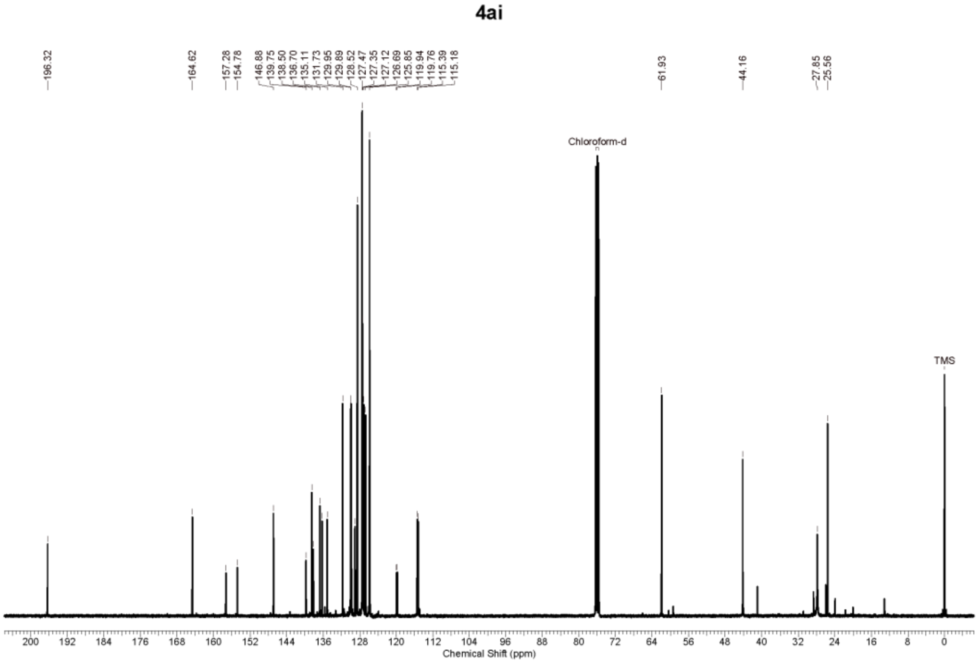

(*E*)-2-Benzyl-4-((4-methoxyphenyl)(4-(trifluoromethyl)phenyl)methylene)-3,3-dimethyl-3,4-dihydroisoquinolin-1(2*H*)-one (4cj)

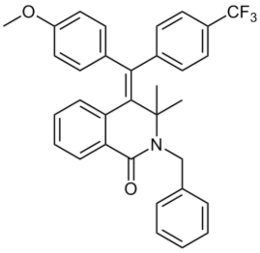

4cj

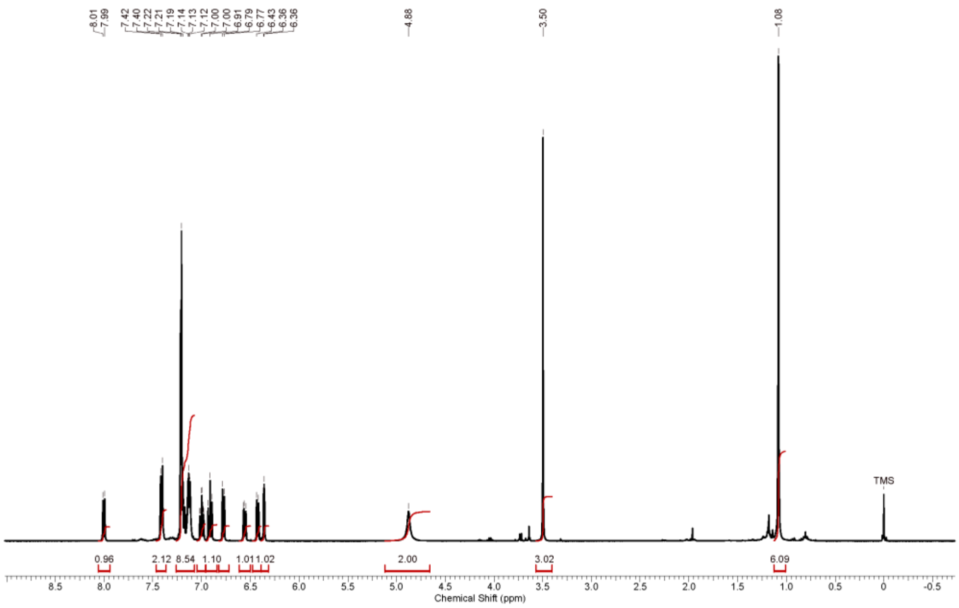

4cj

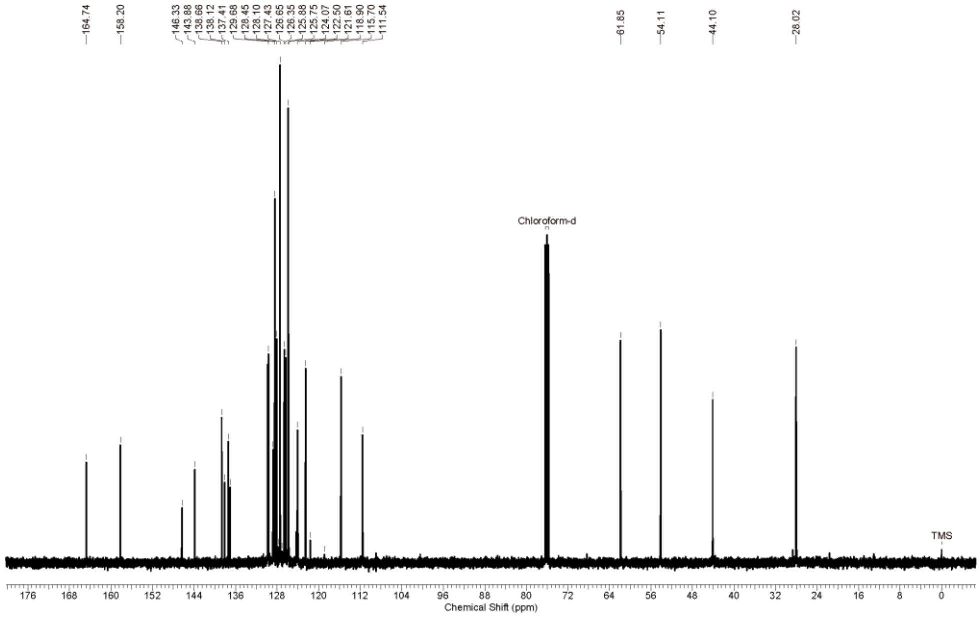

(*E*)-2'-Benzyl-4'-(1-phenylheptylidene)-2',4'-dihydro-1'*H*-spiro[cyclohexane-1,3'-isoquinolin]-1'-one (4da)

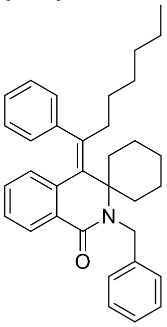

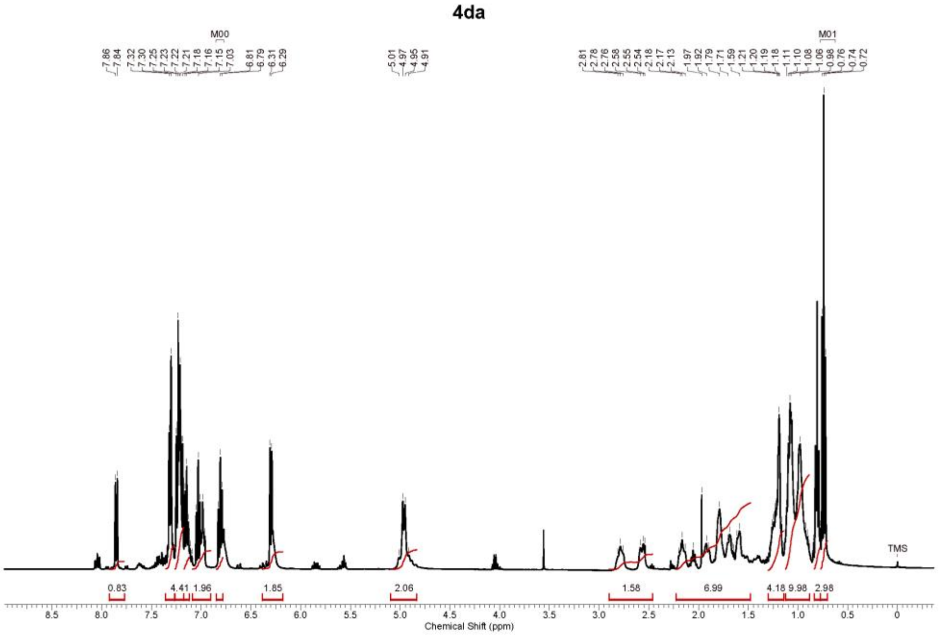

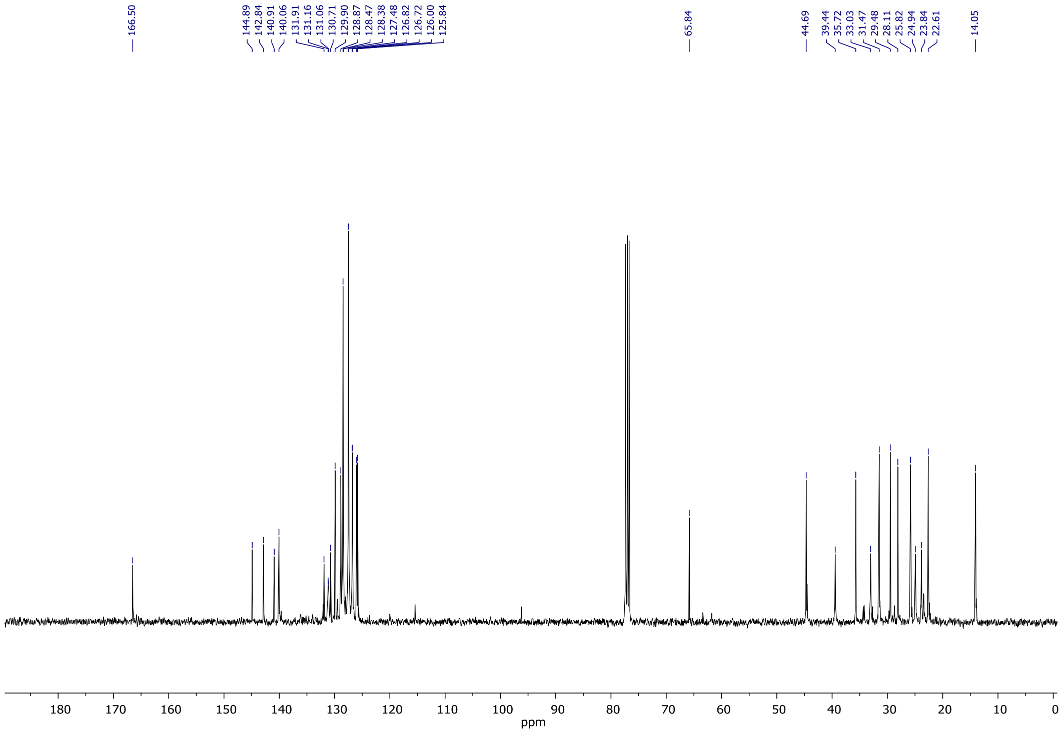

(*E*)-2'-Benzyl-4'-(1-(3-methoxyphenyl)heptylidene)-2',4'-dihydro-1'*H*-spiro[cyclohexane-1,3'-isoquinolin]-1'-one (4db)

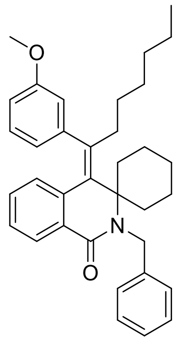

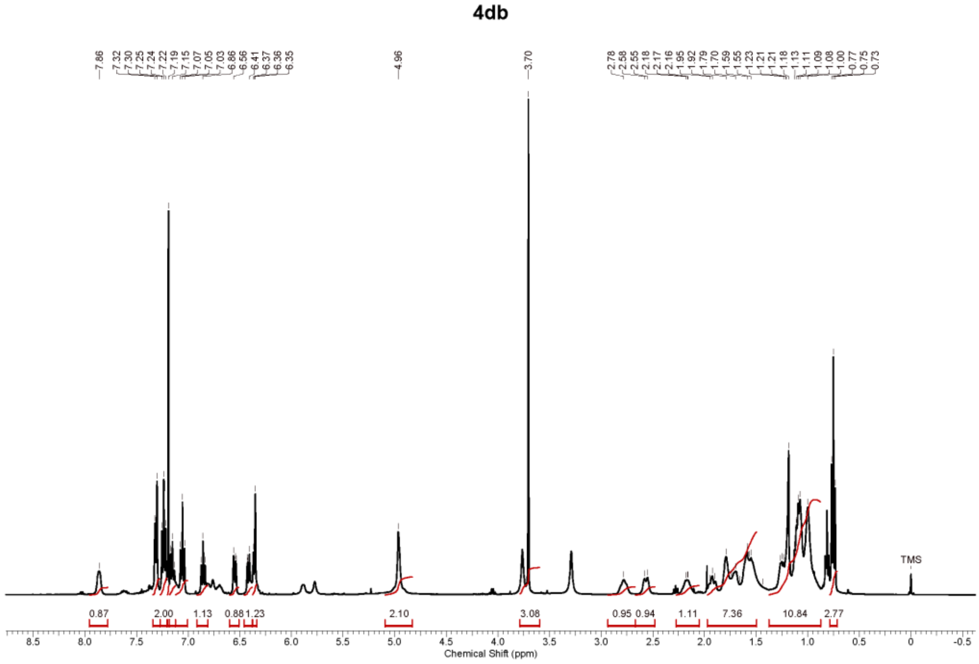

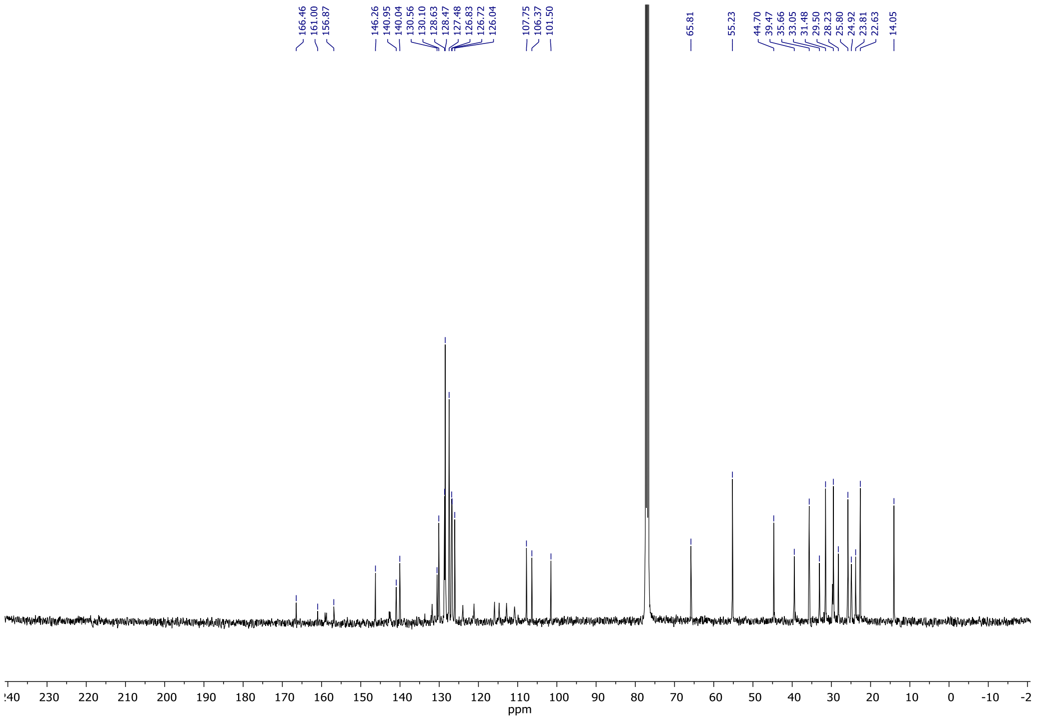

(*Z*)-2-Benzyl-4-((3-methoxyphenyl)(phenyl)methylene)-3-phenyl-3,4-dihydroisoquinolin-1(2*H*)-one (4eb)

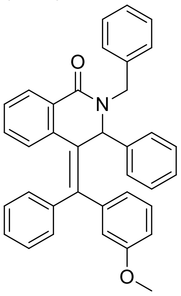

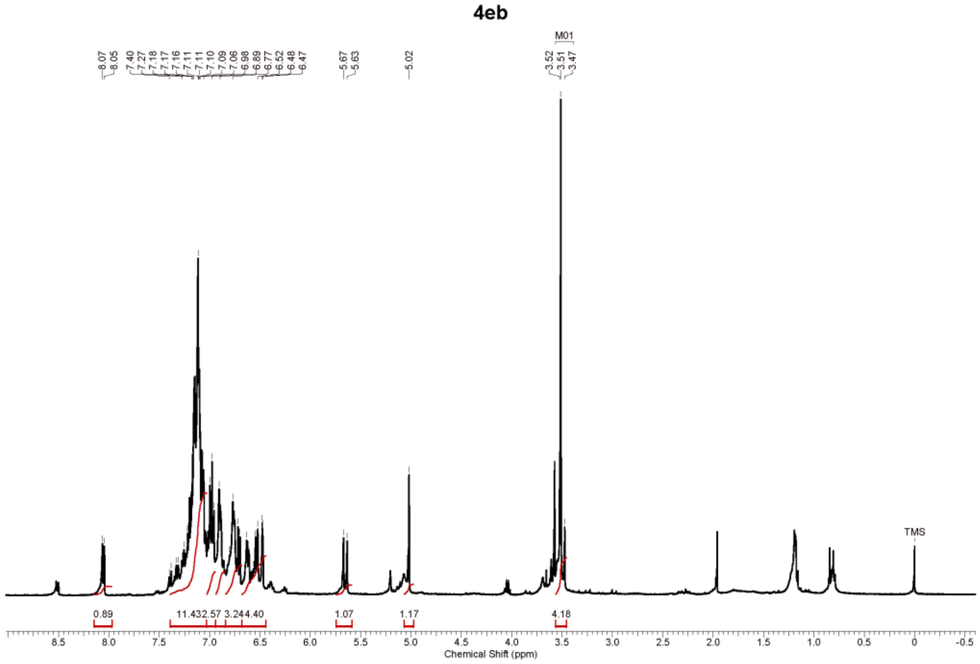

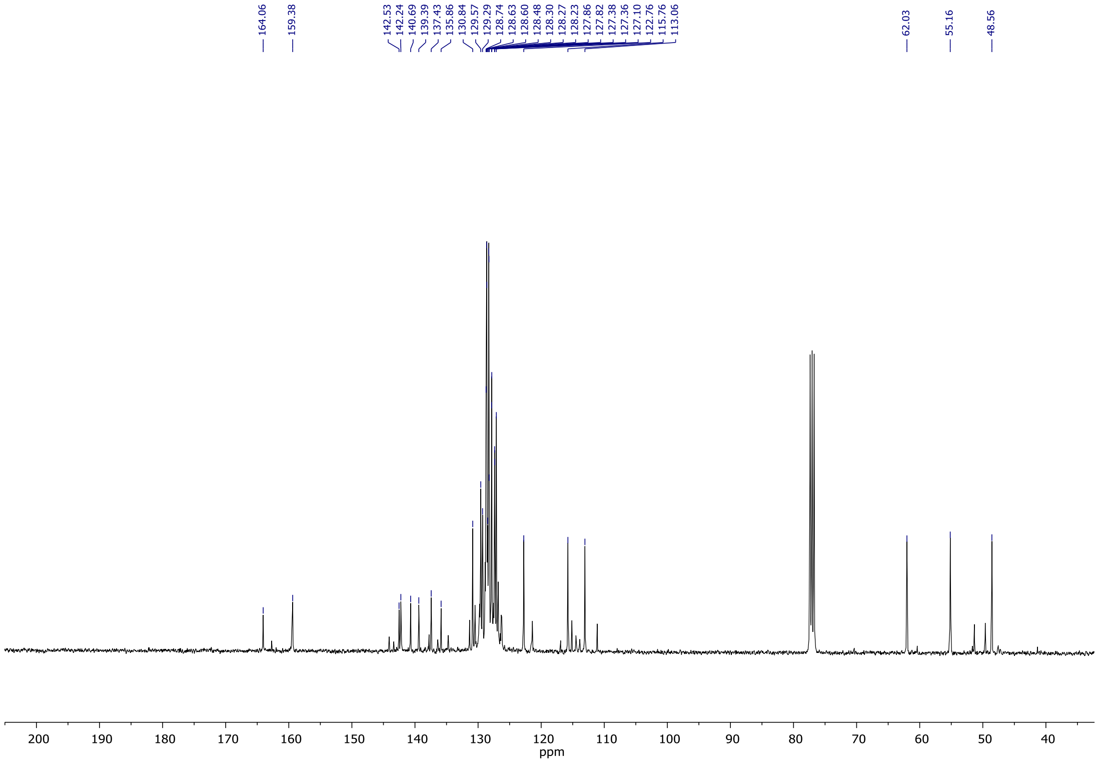

(*E*)-4-((4-Acetylphenyl)(4-fluorophenyl)methylene)-2-benzyl-3,4-dihydroisoquinolin-1(2*H*)-one (4fc)

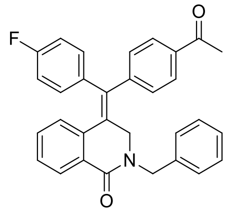

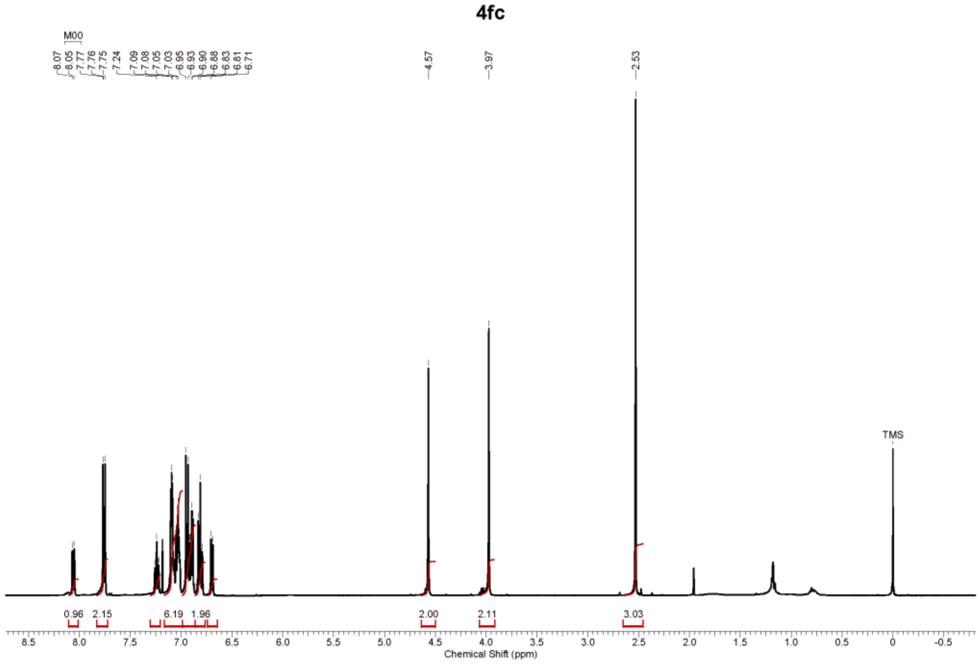

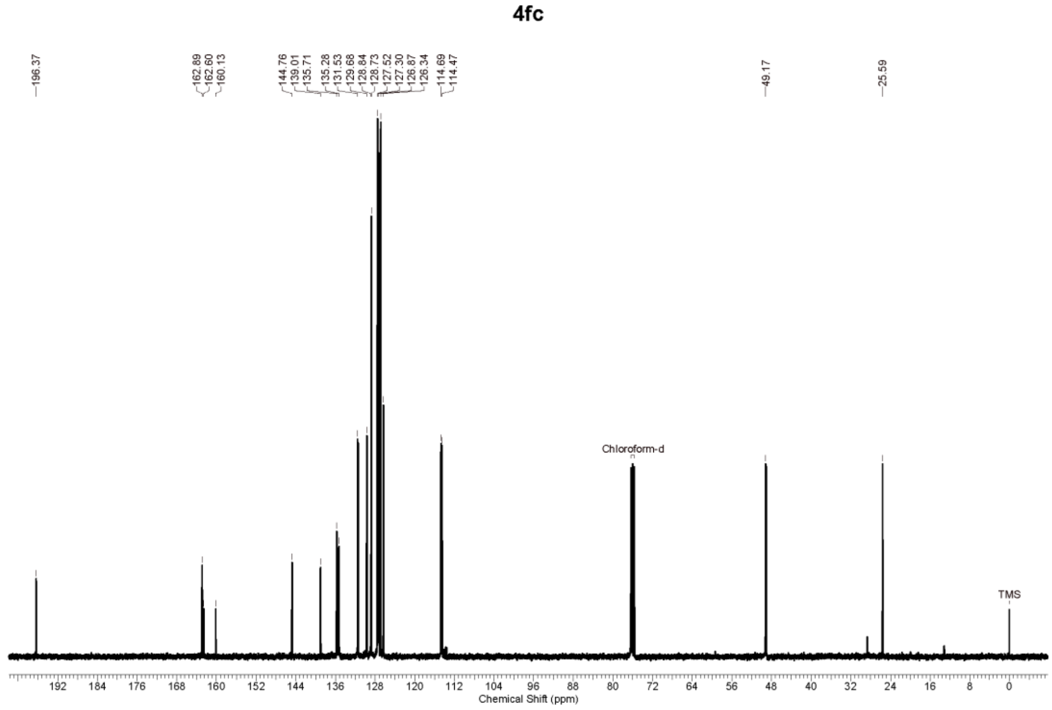

(*E*)-4-((4-Acetylphenyl)(4-methoxyphenyl)methylene)-2-benzyl-3,4-dihydroisoquinolin-1(2*H*)-one (4fj)

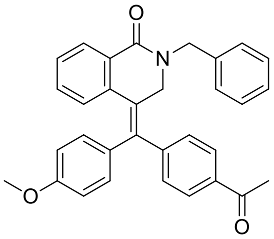

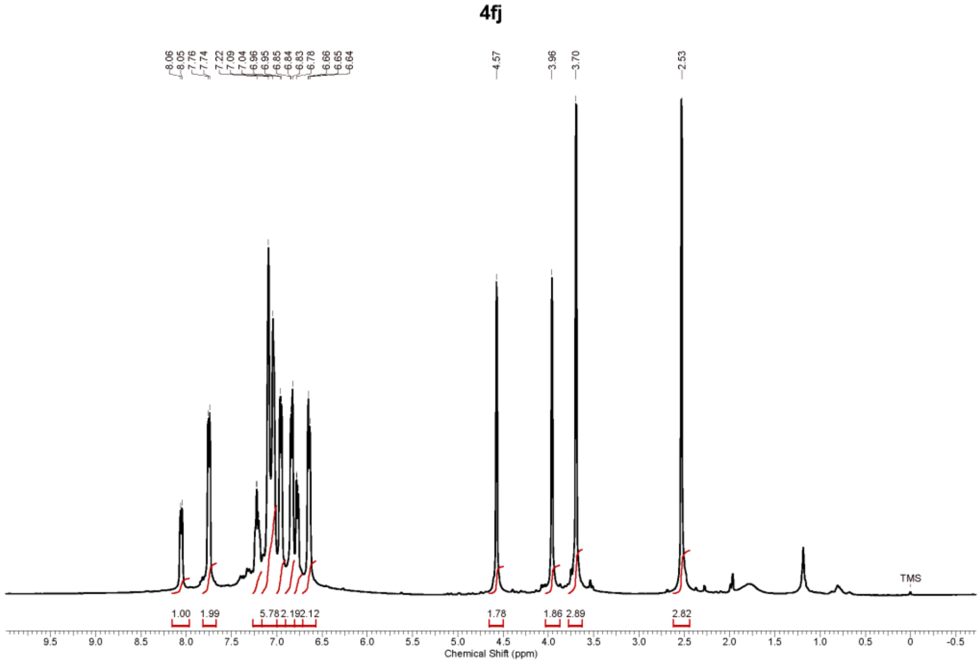

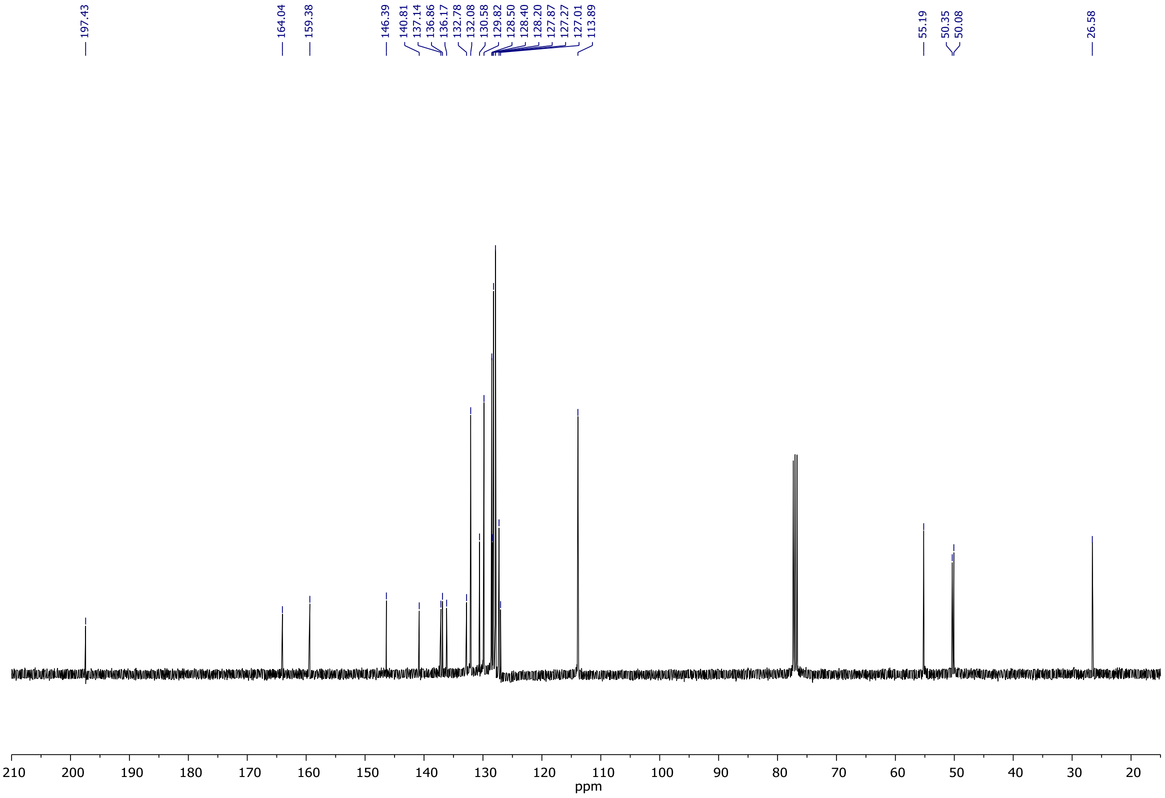

(*E*)-2-Benzyl-4-((4-methoxyphenyl)(2-phenyl-1*H*-indol-3-yl)methylene)-3,3-dimethyl-3,4-dihydroisoquinolin-1(2*H*)-one (6ba)

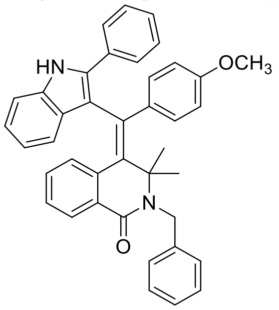

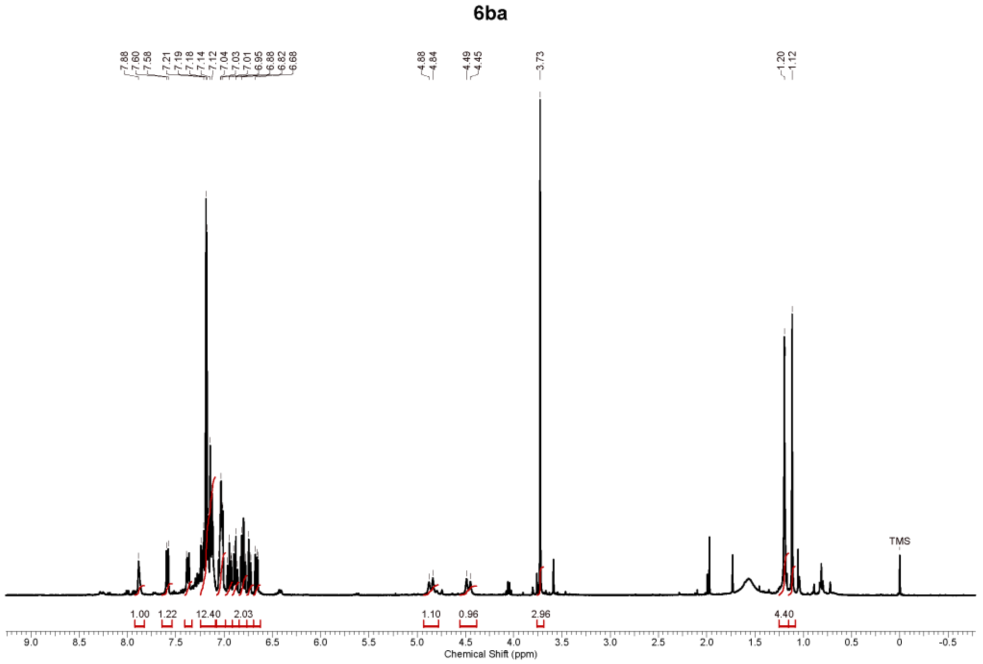

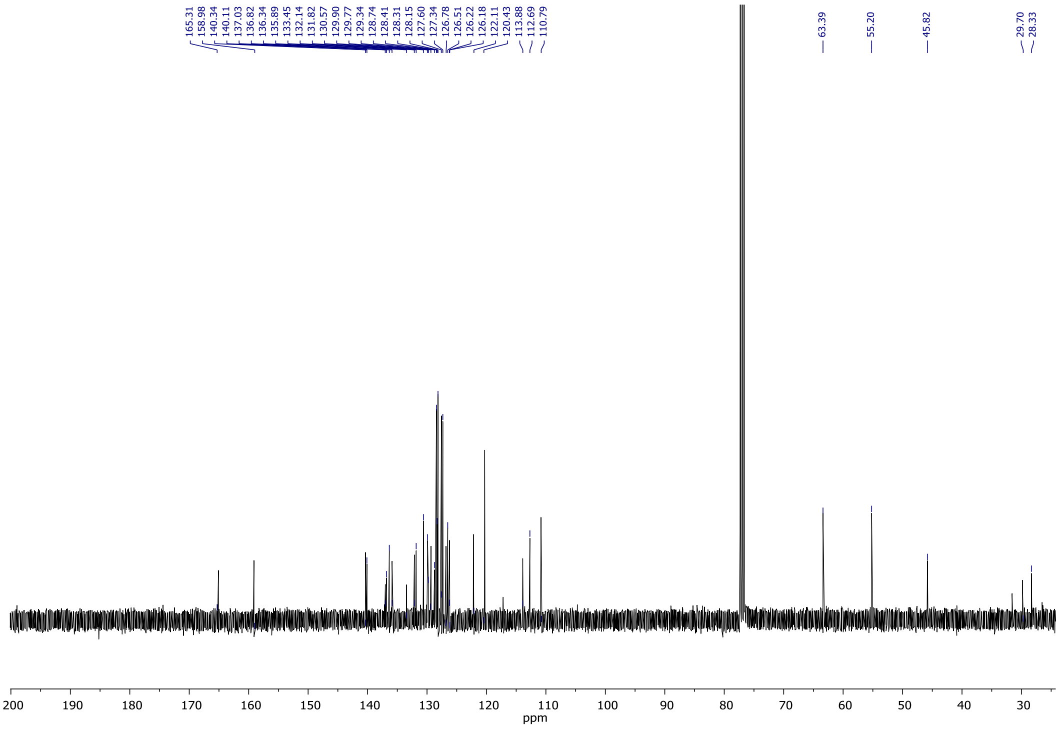

(*E*)-4-((4-Acetylphenyl)(2-phenyl-1*H*-indol-3-yl)methylene)-2-benzyl-3,4-dihydroisoquinolin-1(2*H*)-one (6fa)

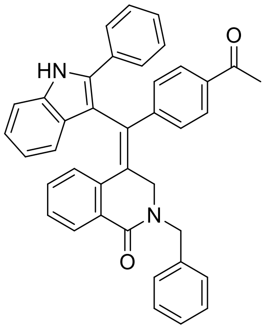

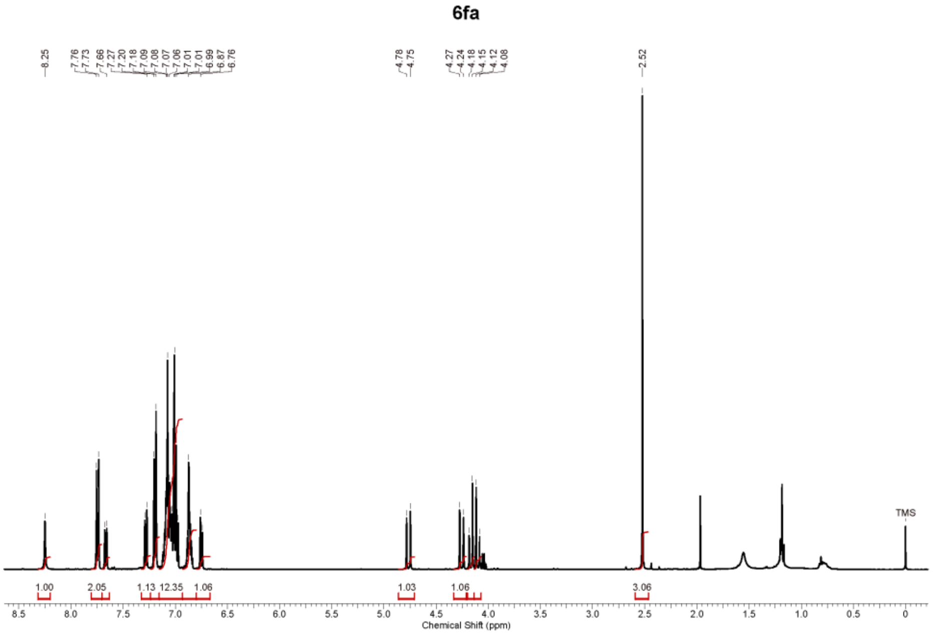

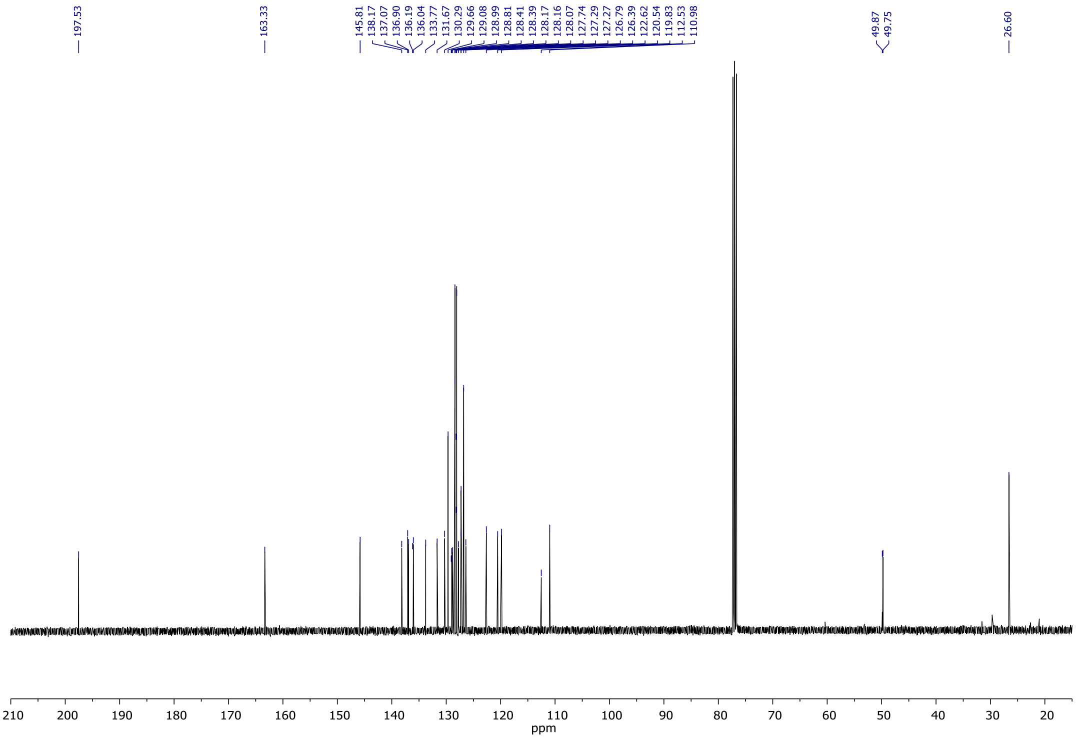

(*E*)-4-((4-Acetylphenyl)(2-(thiophen-2-yl)-1*H*-indol-3-yl)methylene)-2-benzyl-3,4-dihydroisoquinolin-1(2*H*)-one (6fb)

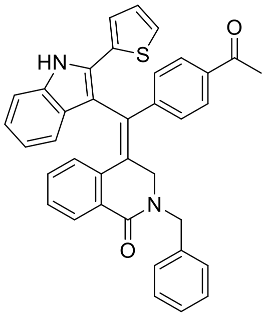

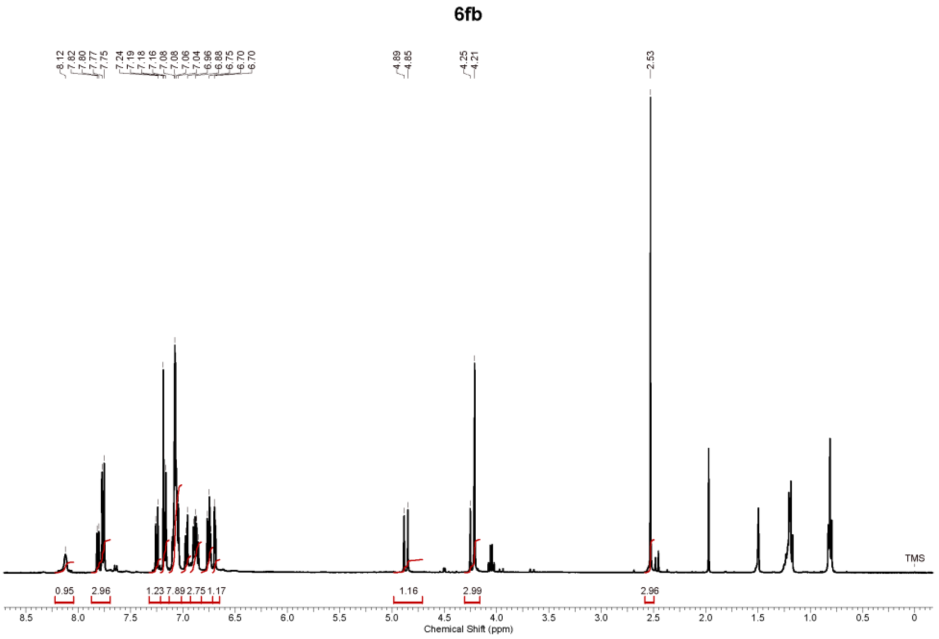

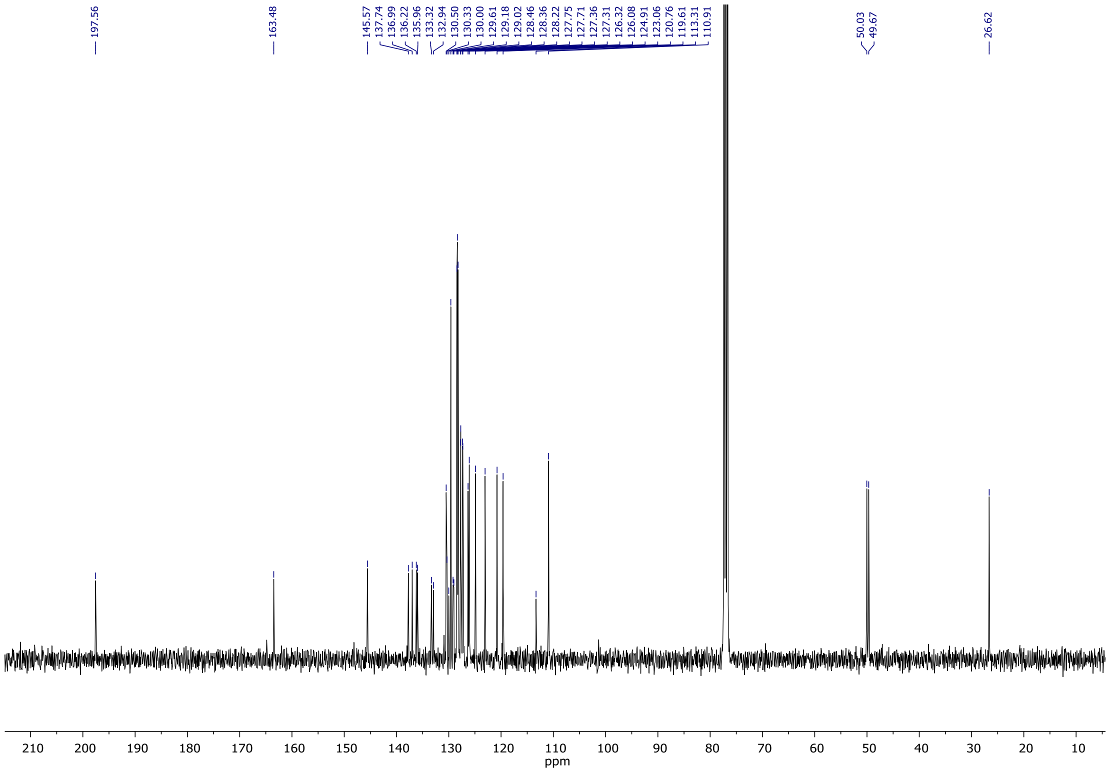

(*E*)-4-((4-Acetylphenyl)(2-((*E*)-cyclooct-1-en-1-yl)-1*H*-indol-3-yl)methylene)-2-benzyl-3,4-dihydroisoquinolin-1(2*H*)-one (6fc)

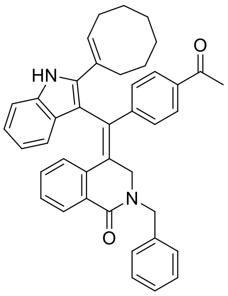

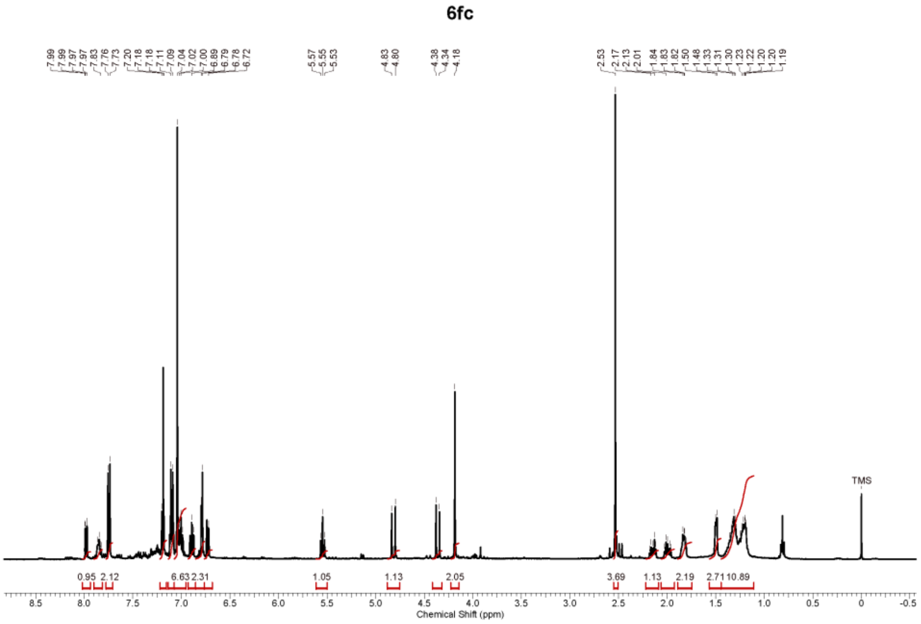

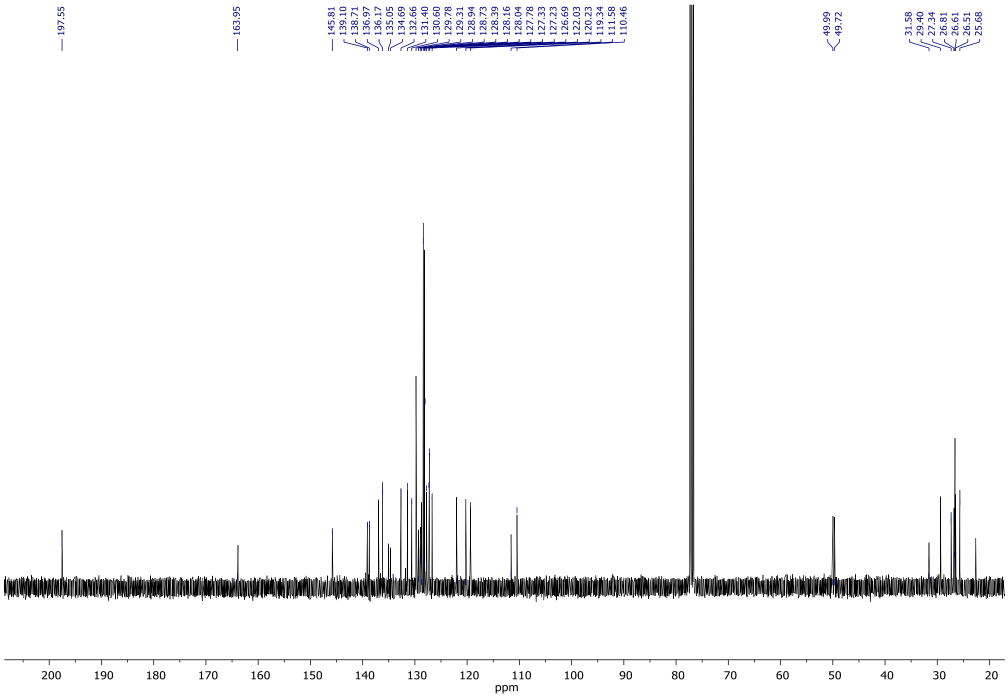

Determination of double bond configuration:

The absolute stereochemistry of the double bonds in **4fj** and **6fa**, as model compounds, was assigned by NOESY experiments, which demonstrated, for both, the *E* configuration.

In order to assing the correct stereochemistry it was necessary to analyze the ^{1}H NMR and ^{13}C NMR signals that were obtained by homonuclear 2D NMR experiments (COSY and NOESY) and heteronuclear 2D NMR experiments (HSQC).

(*E*)-4-((4-Acetylphenyl)(4-methoxyphenyl)methylene)-2-benzyl-3,4-dihydroisoquinolin-1(2*H*)-one (4fj)

Key NOE interactions to establish the absolute stereochemistry of **4fj** were those of proton 7 and methylene 10 (for structure numbering see Figure S1) of dihydroisoquinolone with the substituents 4-methoxyphenyl and 4-acetylphenyl. The NOESY showed, proximity between proton 7 of dihydroisoquinolin-1(2*H*)-one and protons 27,31 of 4-methoxyphenyl and proximity of methylene 10 and protons 21,25 of 4-acetylphenyl (see Figure S1)

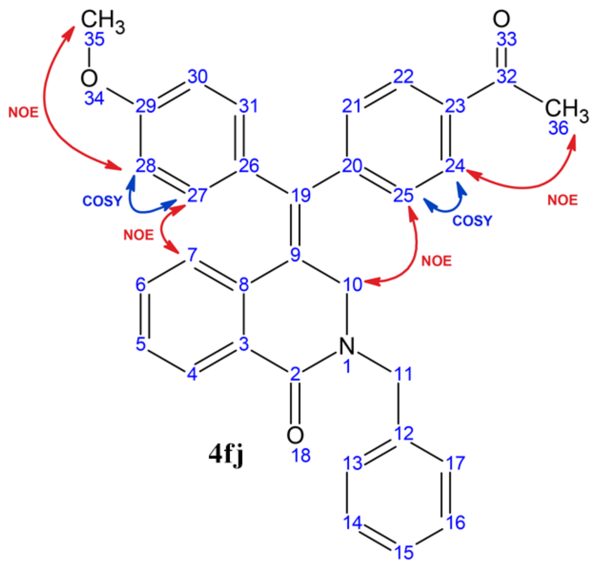

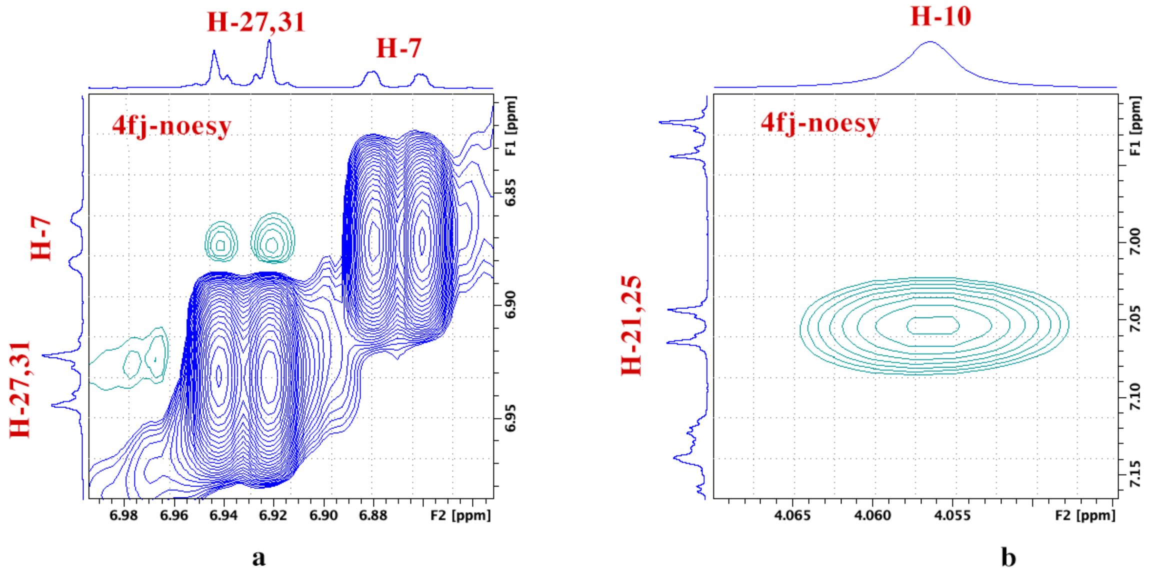

Figure S1: Structural numbering and key NOESY and COSY correlations of **4fj.** (a) Correlation H_1-27,28/H_1-7; (b) correlation H_1-21,25/H_2-10.

(*E*)-4-((4-Acetylphenyl)(2-phenyl-1*H*-indol-3-yl)methylene)-2-benzyl-3,4-dihydroisoquinolin-1(2*H*)-one (6fa)

Key NOE interactions to establish the absolute stereochemistry for **6fa** were those of proton 7 and methylene 10 (for structural numbering see Figure S2) of dihydroisoquinolione with the substituents phenylindolyl and 4-acetylphenyl. The crowded spectra for resonance of phenylindolyl did not allow unequivocal attribution of NOESY signals, instead the proximity between methylene protons, 10, and protons 30,34 of 4-acetylphenyl was clear (see Figure S2).

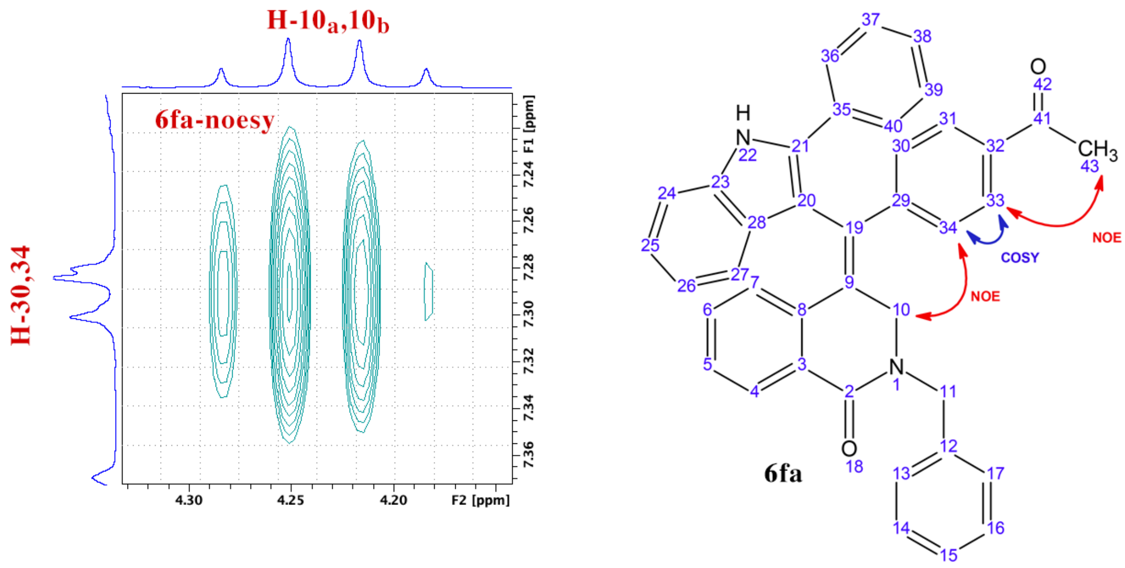

Figure S2: Structure numbering and key NOESY and COSY correlations of **6fb**. correlation H_1-30,34/$H_{a,b}$-10.
